# Morphology-Controlled Growth of Crystalline Ag–Pt-Alloyed Shells onto Au Nanotriangles and Their Plasmonic Properties

**DOI:** 10.1021/acs.jpcc.3c02897

**Published:** 2023-08-03

**Authors:** Xiaobin Xie, Marijn A. van Huis, Alfons van Blaaderen

**Affiliations:** Soft Condensed Matter, Debye Institute for Nanomaterials Science, Utrecht University, Princetonplein 5, 3584 CC Utrecht, The Netherlands

## Abstract

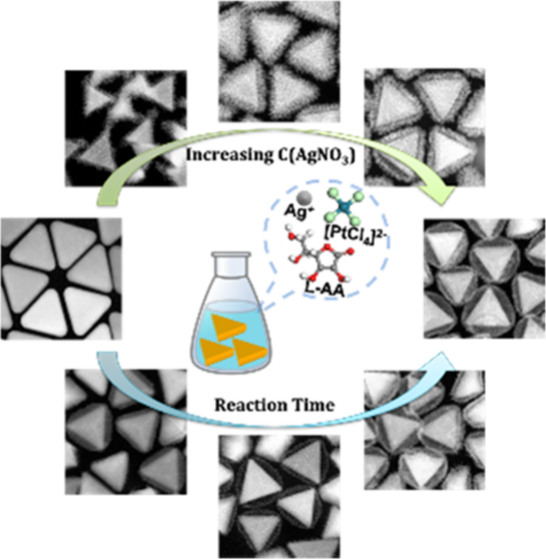

The surface plasmon resonance of noble-metal nanoparticles depends on nanoscale size, morphology, and composition, and provides great opportunities for applications in biomedicine, optoelectronics, (photo)catalysis, photovoltaics, and sensing. Here, we present the results of synthesizing ternary metallic or trimetallic nanoparticles, Au nanotriangles (Au NTs) with crystalline Ag–Pt alloyed shells, the morphology of which can be adjusted from a yolk–shell to a core–shell structure by changing the concentration of AgNO_3_ or the concentration of Au NT seeds, while the shell thickness can be precisely controlled by adjusting the concentration of K_2_PtCl_4_. By monitoring the growth process with UV–vis spectra and scanning transmission electron microscopy (STEM), the shells on the Au NT-Ag-Pt yolk–shell nanoparticles were found to grow via a galvanic replacement synergistic route. The plasmonic properties of the as-synthesized nanoparticles were investigated by optical absorbance measurements.

## Introduction

Gold-based ternary (or trimetallic) noble-metal nanoparticles (TNM-NPs) have attracted great interest due to their promising applications in various fields, including catalysis,^1−6^ energy conversion,^7−10^ and sensing.^11−14^ In the course of decades, a wide variety of TNM-NP structures, like core–shell, alloyed, Janus, yolk–shell, and other complex morphologies have been successfully synthesized.^1^ Nanostructures based on plate-like Au nanoparticles are of particular importance because of their special morphology and highly tunable properties.^15−18^ The localized surface plasmon resonance (LSPR) of Au nanotriangle (Au NT)-based TNM-NPs can be tuned by tailoring their size, composition, and geometric morphology.^19−22^ Furthermore, manipulating the spatial distribution of metals on Au NTs allows fine-tuning of the plasmonic and/or chemical and physical properties of these TNM-NPs for enhanced (photo-) catalysis and Raman scattering.^20,23^

The seed-mediated wet chemical approach is widely used for synthesizing TNM-NPs for realizing applications utilizing plasmonic and catalytic properties.^24−27^ Particularly, the combination of plasmonic metals and catalytically active metals into the same nanoparticle is a versatile way to construct high-performance photocatalysts.^28−30^ However, damping and broadening of the surface plasmons occur as some catalytic metals, e.g., Pt and Pd, are growth onto less-lossy plasmonic metal nanoparticles.^31^ Recent reports indicate that the decay of surface plasmon intensity depends on the thickness of a more-lossy-plasmonic shell of a core–shell nanoparticle, or on structural relationships, such as Janus and yolk–shell structures, composed of better-performing plasmonic and more-lossy-plasmonic metals.^32−34^ Therefore, most of the current investigations have focused on precisely controlled growth of atomic layers,^28^ or on site-selective growth,^6,10,35^ or alloyed Pd and/or Pt onto Au and/or Ag substrates to achieve highly active (photo)catalysts since other structures, e.g., yolk–shell and core frame, are still challenging to synthesize and are therefore hardly explored. Additionally, the addition of higher-temperature melting metals like Pd can increase the shape stability of interesting out-of-equilibrium particle shapes that are interesting for surface-enhanced Raman scattering (SERS).^27^

In the present work, we developed a facile strategy for the morphology-controlled synthesis of TNM-NPs with a yolk–shell structure that are formed by the growth of Ag-Pt alloyed shells onto Au NT cores, which feature an emerging hollow space between the Au NT cores and the Ag-Pt alloyed shells. Furthermore, we found that the structure of the Au NT-Ag-Pt NPs can be tuned from a yolk–shell structure to a core–shell structure by changing the concentration of AgNO_3_ or Au NTs seeds. The thickness of the Ag-Pt shells was found to rely on the concentration of K_2_PtCl_4_ used for the synthesis. Moreover, the growth mechanism, which was revealed by time-dependent UV–vis spectroscopy, scanning transmission electron microscopy–high-angle annular dark-field (STEM-HAADF) imaging, and STEM–X-ray energy-dispersive spectrometry (STEM-EDS) mapping, indicated that the yolk–shell structure formed via a Galvanic replacement-mediated growth path. Finally, how the different shapes and morphologies of these trimetallic NPs affected the plasmonic properties was investigated by UV–vis spectroscopy.

## Methods

### Chemicals

Used chemicals were hydrogen tetrachloroaurate (III) hydrate (HAuCl_4_, 99.9%, Sigma), sodium borohydride (NaBH_4_, 96%, Sigma), silver nitrate (AgNO_3_, ≥99.9%, Sigma), sodium tetrachloropalladium (II) (Na_2_PdCl_4_, 99.95%, Sigma), potassium tetrachloroplatinate(II) (K_2_PtCl_4_, 99.95%, Sigma), l-ascorbic acid (AA, 99.98%, Sigma), cetyltrimethylammonium chloride (CTAC, ≥99%, Sigma), and CTAC solution (25 wt % in water, Sigma-Aldrich). All chemicals were used as received without further purification. Also, deionized water with a resistivity of 18.2 MΩ·cm at 25 °C was used in the experiment.

### Synthesis and Purification of Au NTs

The Au NTs were synthesized by a modification of a previously reported synthesis.^36^ (1) Au seeds@CTAC: 25 μL of 50 mM HAuCl_4_ was mixed with 4.70 mL of 0.10 M CTAC solution in a 20 mL glass vial. Next, 300 μL of fresh prepared 10 mM NaBH_4_ was injected into the above mixture while stirring. The seeds solution was strongly stirred for 2 min. After that, it was kept at room temperature for at least 2 h. After 2 h, the Au seeds solution was diluted 10 times by mixing 0.50 mL of Au seeds and 4.50 mL of 0.10 M CTAC. (2) 1.60 mL of 0.10 M CTAC, 40 μL of 50 mM HAuCl_4_, and 30 μL of 10 mM KI were added into 8.00 mL of deionized water in a 20 mL glass vial one by one. This solution was marked as solution-A. (3) 60 mL of deionized water was added into a 250 mL round-bottom flask. 60 mL of 0.10 M CTAC, 1.5 mL of 50 mM HAuCl_4_, and 900 μL of 10 mM KI were injected into the deionized water. This solution was marked as solution-B. (4) 40 μL and 1.20 mL of 0.10 M AA solution were injected into solution-A and solution-B, respectively, while stirring. As both solution-A and solution-B turned colorless, 150 μL of diluted Au seeds was injected into solution-A. Stirring was continued for about 1 min. All solution-A was added into solution-B while stirring. After the two solutions were well mixed, it was left undisturbed for about 2 h, which allows growth of the Au nanocrystals. After allowing growth for about 2 h, 34.0 mL of the above growth solution and 6.50 mL of 25 wt % CTAC were mixed in a 50 mL centrifuge tube, and then left undisturbed for 12 h. The supernatant was removed carefully, and the sediment was resuspended in 35.0 mL of 10 mM CATC and served as a stock solution for further use.

### Synthesis of Au NT-AgPt NPs

Typically, 7.40 mL of deionized water, 1.00 mL of 0.10 M CTAC solution, and 1.50 mL of Au NTs stock solution (λ_max_ = 645 nm and maximum extinction of 0.64 when diluted three times in H_2_O) were mixed in a 20 mL glass vial, next 50 μL of 10 mM AgNO_3_, 50 μL of 10 mM K_2_PtCl_4_, and 100 μL of 0.10 M AA were added to the above mixture with magnetic stirring. It was left to react at a temperature of 20 °C for 15 h. The product was collected by centrifugation at 8000 rcf (relative centrifugal force) for 10 min (Eppendorf Centrifuge 5424 R) and then washed with deionized water once, to remove excess CTAC. More details and the corresponding data are shown in Tables S1 and S2.

### Synthesis of Au NT-AuAgPt NPs

Typically, 7.40 mL of deionized water, 1.00 mL of 0.10 M CTAC solution, and 1.50 mL of Au NTs stock solution (λ_*max*_ = 645 nm and maximum extinction of 0.64 when diluted three times in H_2_O) were mixed in a 20 mL glass vial, next 40 μL of 10 mM HAuCl_4_, 20 μL of 10 mM AgNO_3_, 30 μL of 10 mM K_2_PtCl_4_, and 100 μL of 0.10 M AA were added to the above mixture with magnetic stirring. It was left to react at room temperature for 15 h. The product was collected by centrifugation at 8000 rcf (relative centrifugal force) for 10 min (Eppendorf Centrifuge 5424 R) and then washed with deionized water once, to remove excess CTAC. More details and the corresponding data are shown in Table S3.

### Synthesis of Au NT-AgPd NPs

7.40 mL of deionized water and 2.50 mL of Au NTs stock solution (λ_max_ = 645 nm and maximum extinction of 0.64 when diluted three times in H_2_O) were added to a 20 mL glass vial, after which 50 μL of 10 mM AgNO_3_, 50 μL of 10 mM Na_2_PdCl_4_, and 100 μL of 0.10 M AA were added into the above mixture with magnetic stirring. After reacting at 20 °C for 15 h, the product was collected by centrifugation at 8000 rcf (relative centrifugal force) for 10 min (Eppendorf Centrifuge 5424 R) and washed with deionized water once, to remove excess CTAC. More details and the corresponding data are shown in Table S4.

### Characterization

Ultraviolet–visible (UV–vis) spectroscopy was performed with a Lambda 750 UV–Vis spectrometer (PerkinElmer). Bright-field transmission electron microscopy (TEM) images and scanning transmission electron microscopy–high-angle annular dark-field (STEM-HAADF) images were acquired with an FEI Talos F200X operating at 200 kV while using a camera length of 98 mm and equipped with a ChemiSTEM EDS detector used for STEM-EDS chemical mapping.

## Results and Discussion

The Au NTs were synthesized by modifying a previously reported protocol.^36^ In Figure S1, the TEM and STEM images clearly show the triangular platelet shape of the Au NTs where the edge length is about 63 nm and the thickness is about 22 nm. The main localized surface plasmon resonance (LSPR) band of the Au NTs is 640 nm (Figure S1e). Using as-synthesized Au NTs as seeds, we performed experiments on overgrowing of Ag and Pt in a single growth step onto the plate-like Au nanocrystals by co-reducing AgNO_3_ and K_2_PtCl_4_ with l-ascorbic (AA) while using CTAC as a surfactant/ligand.

**Figure 1:**
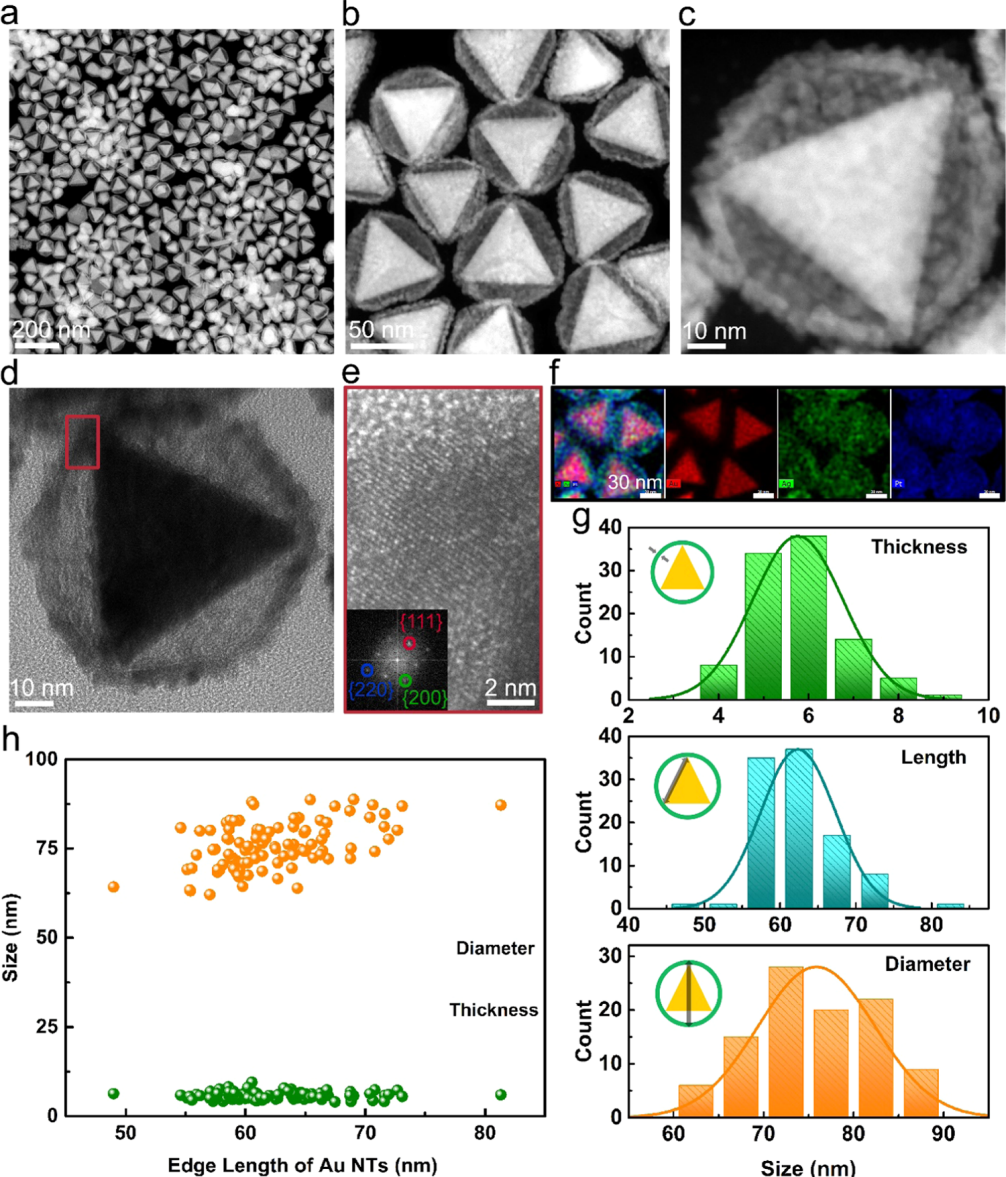
Morphology and structure of Au NT-AgPt nanoparticles. (a–c) STEM-HAADF images of Au NT-AgPt NPs, (d, e) HRTEM images of Au NT-AgPt NPs, (f) STEM-EDS elemental maps of Au NT-AgPt NPs, the scale bar indicates 30 nm. (g) Thickness, edge length, and diameter distribution of Au NT-AgPt NPs. (h) Relationship between edge length and diameter, and between edge length and thickness.

The typical morphology of the Au NT-Ag-Pt NPs is shown in Figure 1. The STEM-HAADF images (Figure 1a–c) show that a porous shell had formed, covering the Au NT core, and was connected to the core at the three corners of the Au NT. The hollow space between the shell and the Au NT core can be clearly observed in these images. The TEM image (Figure 1d) of an individual Au NT-Ag-Pt nanoparticle corroborates the findings in the STEM-HAADF images. Moreover, the atomic lattice fringes in the high-resolution TEM (HRTEM) image of the shell are 0.20, 0.23, and 0.14 nm, which are close to the {200}, {111}, and {220} interplanar fcc spacings between Ag (*d*(200) = 0.204 nm, *d*(111) = 0.235 nm, and *d*(220) = 0.144 nm) and Pt (*d*(200) = 0.198 nm, *d*(111) = 0.229 nm, and *d*(220) = 0.140 nm),^38^ respectively. To resolve the distribution of elements in the nanoparticles, STEM-EDS (X-ray energy-dispersive spectrometry) mapping was conducted. As we expected, the porous shell is composed of alloyed Ag-Pt, while the triangular core consists of Au (Figure 1f). The size of the Au NT-Ag-Pt nanoparticles was determined by measuring the dimensions of 100 nanoparticles from the STEM images (Figure 1a). The average thickness of the Ag–Pt shell was ∼5 ± 1 nm, the average edge length of Au NT cores was ∼63 ± 5 nm, and the average diameter of the whole particles was ∼77 ± 6 nm (Figure 1g). There was a weak positive correlation between the diameter of the nanoparticles and the edge length of Au NTs core, and there was no correlation between the shell thickness and the diameter of whole particles or with the edge length of the Au NTs (Figure 1h). Moreover, considering the thin Ag–Pt shell, it may collapse sometime after the synthesis. To uncover the structural stability, we checked the same TEM sample which was stored at room temperature for 6 years. As shown in Figure S2, part of the Ag–Pt shell on NPs totally collapsed, and tiny particles were found around them. However, the structure profile of Au NT-Ag-Pt yolk–shell NPs was still clearly recognized in many of the particles.

To gain insight into the role of reaction species concentrations, including the concentrations of the Au NTs, Ag^+^, [PtCl_4_]^2–^, and AA, on the growth mechanism and the morphologies of these trimetallic nanocrystals, a series of experiments were carried out by accurately controlling the reacting species concentrations and conditions. As shown in Figure S3a–d, the hollow space between the core and the Ag–Pt shells could be tuned by changing the concentration of the Au NTs, where an increase thereof resulted in lowering the volume of the hollow spaces between the cores and the shells (experimental details are provided in Table S1: 8–11). It is well known that the plasmonic properties of metallic nanoparticles strongly depend on their morphology, size, and composition. The LSPR band of the Au NT-Ag-Pt NPs having different hollow spaces were measured by a UV–vis spectrophotometer and are shown in Figure S3e,f. In comparison to the Au NTs, the LSPR band of all particles became broader while the intensity was weaker. For those nanoparticles shown in Figure S3a–c, which are yolk–shell nanostructured particles, their band positions shifted to shorter wavelengths, and the band position of the core–shell particles in Figure S3d showed a red shift.

**Figure 2:**
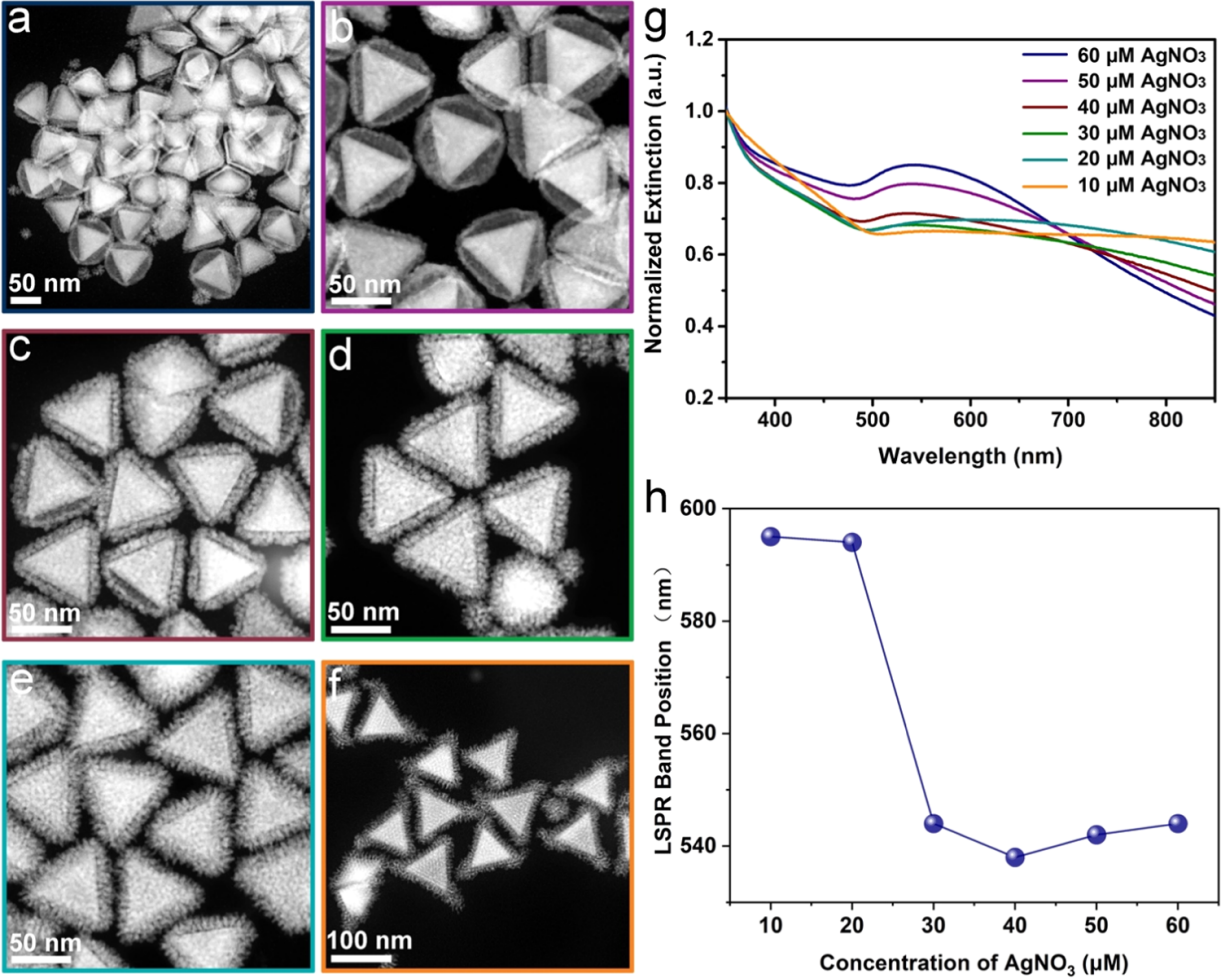
STEM-HAADF images and UV–vis spectra showing the morphological evolution and plasmonic properties of the Au NT-Ag-Pt NPs varying with the concentration of AgNO_3_ used in the synthesis. The concentration of AgNO_3_ in the growth solution was: (a) 60 μM, (b) 50 μM, (c) 40 μM, (d) 30 μM, (e) 20 μM, (f) 10 μM. More details of the experimental conditions are shown in Table S1 (rows 2–7). (g) UV–vis spectra of Au NT-Ag-Pt NPs. (h) Plot of the LSPR band positions of Au NT-Ag-Pt nanoparticles.

In addition, when reducing the Ag concentration of the synthesis from 60 to 10 μM, the hollow space diminution took place to the point where more conventional Au NT@Ag-Pt core–shell nanoparticles were obtained (Figure 2a–f). In this case, the LSPR band of the nanoparticles showed the same trend as when the concentration of the Au NTs was increased, except that in this case, all of the band positions were blue-shifted by contrast with the LSPR band of Au NTs (Figure 2g,h). Interestingly, in Figure 2f, the density of the Ag–Pt shells at the triangle corners seemed somehow higher than at other parts of the Au NT, which is in good agreement with previous reports that Ag NO_3_ can be used for the site-selective growth of Pt on Au nanoparticles.^6,10,35^

To understand this better, more experiments were performed, where the concentration of AgNO_3_ and K_2_PtCl_4_ was decreased. As shown in Figure S4, a smooth Ag shell formed and covered the whole Au NT if no K_2_PtCl_4_ was used for the synthesis (Figure S4a), and only very tiny Pt clusters with a diameter of about ∼1 nm were found to anchor at the Au NT surfaces (Figures S4d and S5), when no Ag^+^ was added to the synthesis. When using 10 μM AgNO_3_ in the growth, the thickness of the Ag-Pt shell decreased from 20 nm to 5 nm as the concentration of K_2_PtCl_4_ reduced from 50 to 10 μM (Figure S4b,c). The concentration of [PtCl_4_]^2–^ (and not the concentration of Au NTs or Ag) was found to be the most important factor determining the thickness of Ag–Pt shell for yolk–shell nanoparticles. The thickness of Ag–Pt shell was decreasing from ∼5.1 ± 1.2 to ∼4.0 ± 0.7 nm when the concentration of [PtCl_4_]^2–^ reduced from 50 to 10 μM (Figures S6a–d and S7). Moreover, when reducing both the concentration of Ag^+^ and [PtCl_4_]^2–^, Au NT@Ag-Pt nanoparticles were obtained with a shell that was made up of tiny Ag-Pt clusters (Figure S8a–d). The thickness of the Ag-Pt shell was found to affect the plasmonic property of the core–shell and yolk–shell particles in various ways. The main LSPR band position of the Au NT-Ag-Pt core–shell nanoparticles changed following the shell thickness (Figure S8e,f), but the band peaks of yolk–shell nanoparticles were at the same position (Figure S6e,f). The intensity of all LSPR bands became higher as the thickness of the Ag-Pt shell reduced, in both core–shell and yolk–shell nanoparticles. Lastly, we inspected the effects of changing the concentration of AA from 500 to 20 mM (Table S1: 24–27). The results indicated that there was no significant influence of the concentration of AA on the shape of the products for these concentrations (Figure S9).

With inspiration from previous reports on iodide ions, (I^–^) could selectively adsorb on Au {111} face and result in the synthesis of Au Nanoplatelets.^36,39,40^ We further conducted a synthesis of Au NT-AgPt by adding different amounts of KI. As shown in Figure S10, Pt site-selective growth was found when using 60 and 30 μM KI in the growth (Figures S10a,b and S11). Au NT-Ag-Pt core–shell-cluster NPs were acquired when the concentration of KI used for synthesis was reduced to 15, 10, and 5 μM (Figures S10c–e and S12). Under similar conditions, Au NT-AgPt core–shell NPs were obtained with no KI added for synthesis (Figure S10f). The LSPR band of site-selective growth Au NT-AgPt NPs (∼660 nm) showed a slight red shift, and the band peaks of Au NT-Ag-Pt core–shell-cluster (∼580 to 590 nm) were blue-shifted compared to the one of Au NTs (Figure S10g,h).

**Figure 3:**
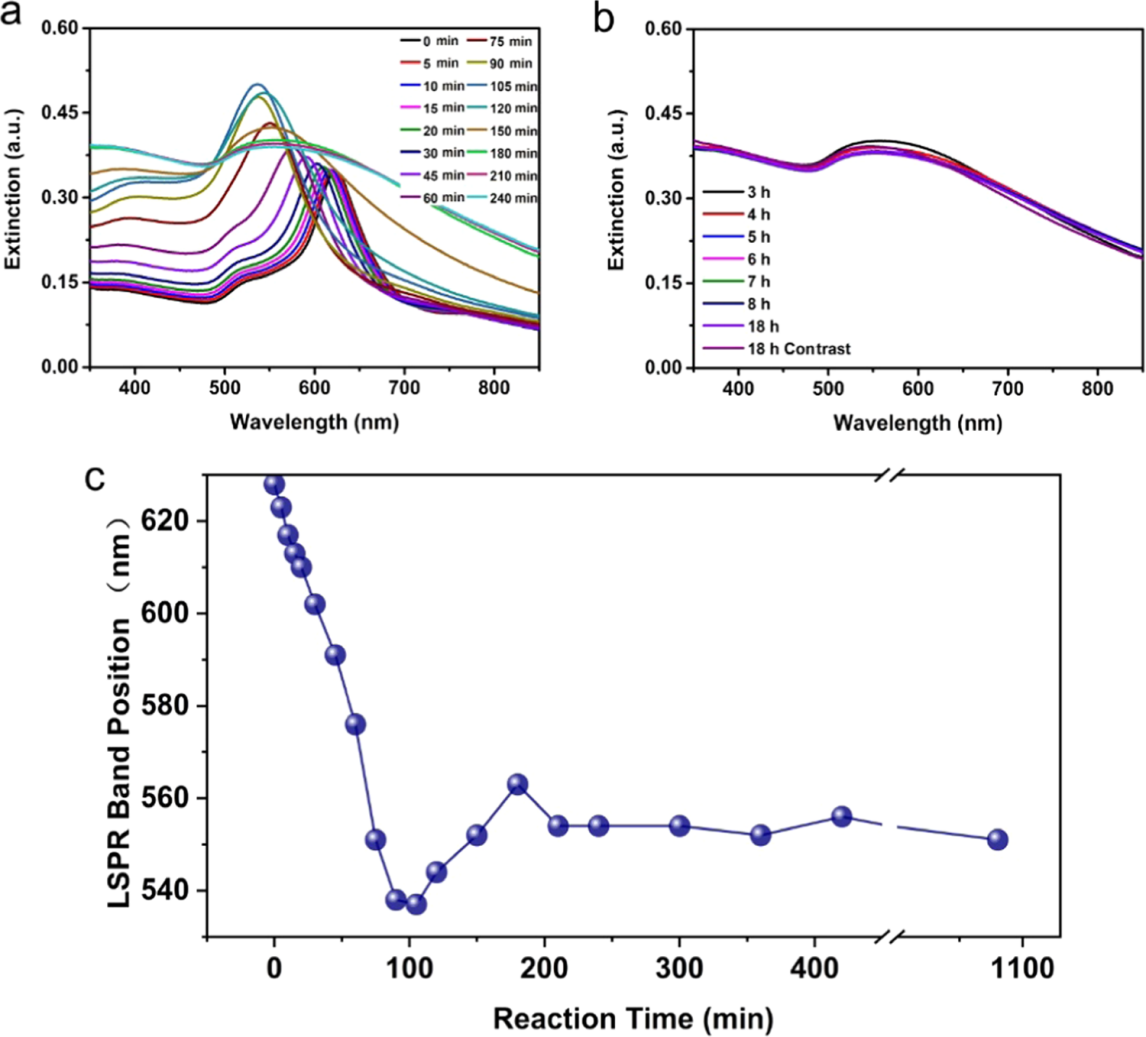
Evolution of the LSPR band during the growth process of Au NT-Ag-Pt NPs. (a, b) UV–vis spectra of the formation of Au NT-Ag-Pt NPs; (c) plot of LSPR band position. The synthesis details are shown in the first row in Table S1.

Because of the dependence of the growth process on the synthesis parameters as discussed above, and the previous reports on the role that Ag plays in the morphology-controlled synthesis of Au-Ag-Pt TMNNPs,^6,35^ we assume that the formation and growth of the yolk–shell structures followed a Galvanic replacement synergistic path, namely, first the more Ag grew onto the Au NT cores followed by the Pt replacing part of the Ag shell, which resulted in the formation of the hollow space between Au NT core and Ag-Pt alloyed shell due to the Kirkendall effect.^41^ To verify this hypothesis, we monitored the growth process in time by recording UV–vis spectra and STEM images at various stages of the growth process. The reaction was stopped by centrifugation after specific reaction times after which UV–vis and TEM measurements were conducted. The UV–vis spectra of the growth process are shown in Figure 3. The LSPR band position was seen to shift dramatically from ∼628 to ∼536 nm in 120 min, moved to ∼546 nm at the 210th min, and retained approximately at this last value till the end of the measurements. In addition to these shifts, the peak became broader in the first 180 min, after which the shape did no longer change.

**Figure 4:**
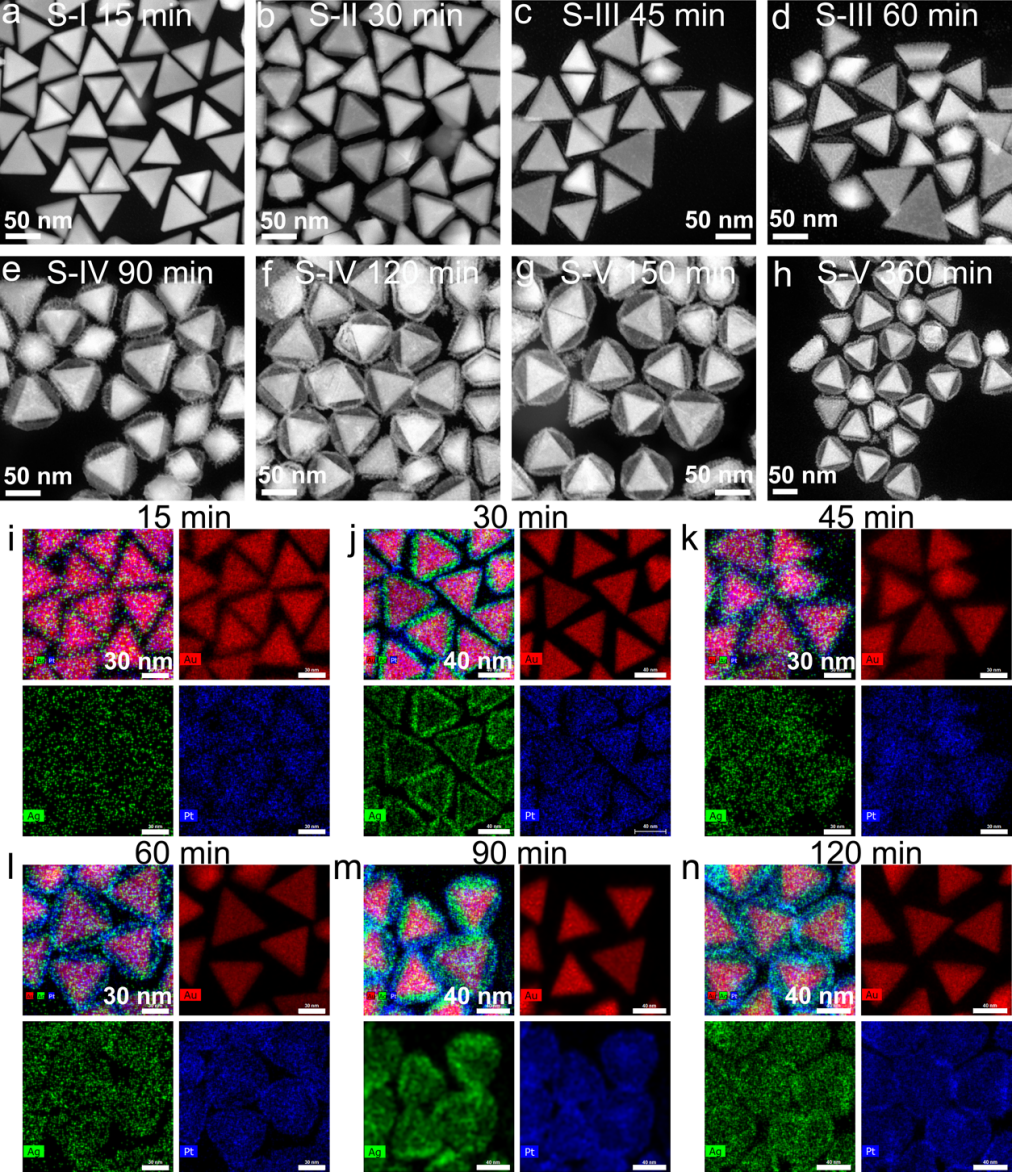
Morphological evolution of Au NT-Ag-Pt NPs with reaction time. (a–h) Typical shape after the specified reaction times; (i–n) STEM-EDS elemental maps of several representative shapes. The synthesis details are shown in the first row in Table S1.

To investigate the morphological evolutions in more detail, STEM-HAADF images were recorded after specific reaction times. According to the STEM images and chemical element mapping images captured at different time-dependent reaction stages, five representative stages were identified in the Au NT-AgPt nanoparticle growth process (Figure 4). At the initial stage (S-I, about 0–20 min), Ag^+^ and [PtCl_4_]^2–^ were reduced and grew onto the Au NTs as an alloyed layer. As the reducing rate of Ag^+^ is faster than that of [PtCl_4_]^2–^,^6,42−44^ at the second stage (S-II, about 21–40 min), Au NT-Ag-Pt core–shell-shell nanoparticles were formed. As the Ag^+^ was consumed, its reduction rate went down, and the Galvanic replacement reaction between Ag and [PtCl_4_]^2–^ became the dominant reaction, resulting in the formation of an Au NT-Ag-Pt core-frame structure, which we identify as the third stage (S-III, about 41–60 min). In the fourth stage (S-IV, about 61–120 min), after the Ag shell on Au NTs was partly replaced by Pt and Ag^+^ ions were dissolved into the solution, where they were reduced by AA again and deposited in the hollow space between Pt and Au NTs. Nevertheless, most of the Ag between the outside shell and the core was finally replaced by Pt and grew into the Au NT-Ag-Pt yolk–shell structure at the end of the final stage (S-V, about 121–360 min).

The chemical oxidation/reduction reaction equations between AA, Ag^+^, and [PtCl_4_]^2–^ are as follows:^45^
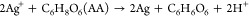

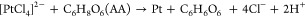

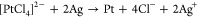
The hydroxyl groups in AA (Figure S13) served as the reacting group and took part in the reactions with the metal precursors.^44^ In addition, the reactivity between Pt precursors and Ag metal is much higher than the reaction rate between AA and [PtCl_4_]^2–^, which is also confirmed by previous works.^6,10^ For this reason, the yolk–shell structure would likely not be formed if AgNO_3_ was partially displaced by HAuCl_4_, which has a higher reaction activity with AA, but the resulting metal—Au—is not easily replaced by [PtCl_4_]^2–^, or [PtCl_4_]^2–^ ions are substituted by [PdCl_4_]^2–^, which has fast reaction rate with AA. Two sets of experiments (first, AgNO_3_ was partially displaced by HAuCl_4_; second, using [PdCl_4_]^2–^ instead of [PtCl_4_]^2–^) were conducted to further verify the above assumption (experimental details are shown in Tables S3 and S4). In agreement with our expectation that yolk–shell particles would not be found, the products in both cases were Au NT-Au-Ag-Pt nanoparticles and Au NT-Ag-Pd nanoparticles containing core–shell structural nanoparticles only (Figures S14 and S15). The Galvanic replacement between Ag and [PtCl_4_]^2–^ caused the Kirkendall effect to occur, which resulted in the formation of the hollow/porous structures, and was initially derived from different diffusion rates between Ag and Pt metals.^46^

**Figure 5:**
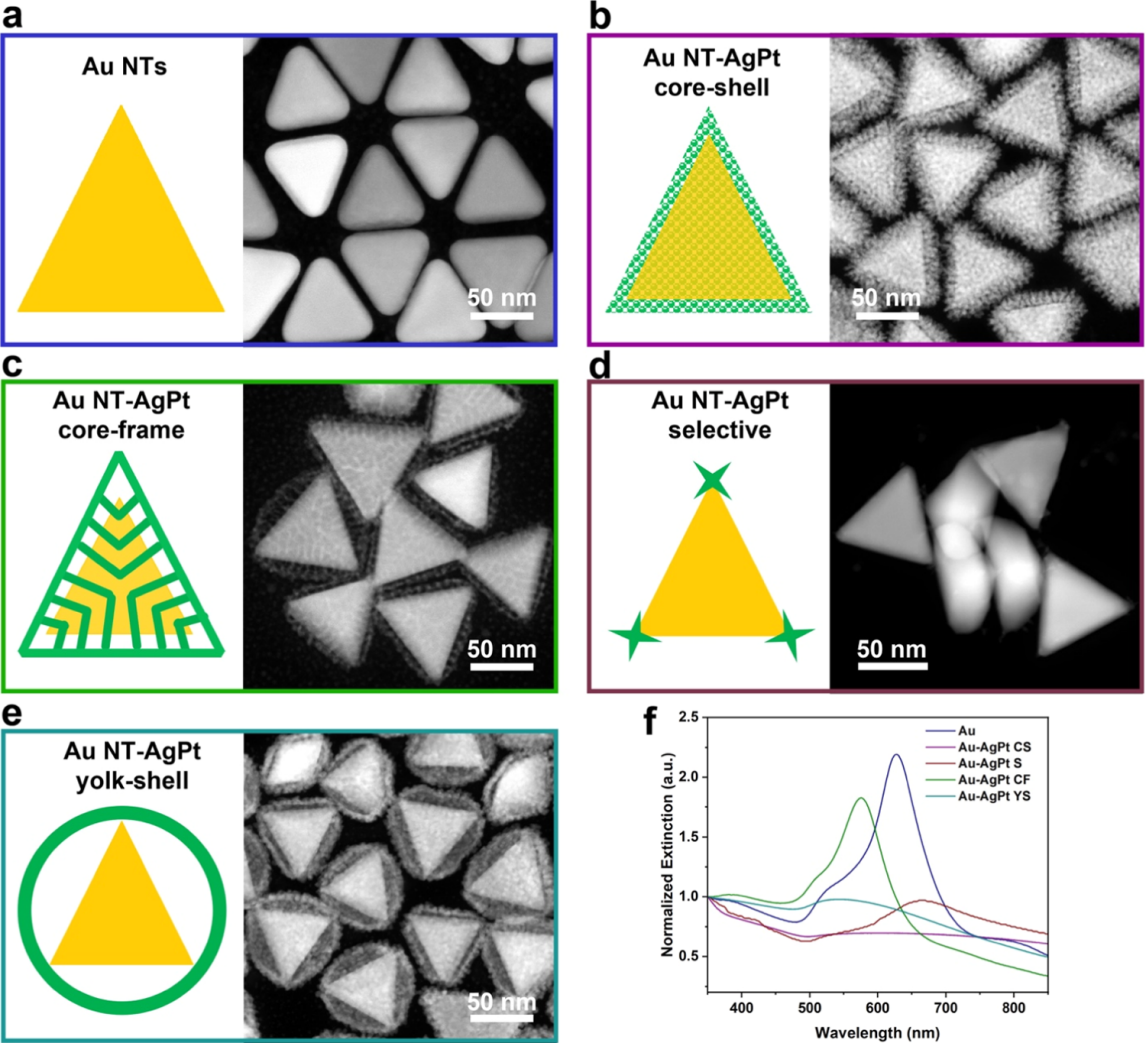
Morphologies and plasmonic properties of several typical Au-Ag-Pt nanoparticles. Geometric model and STEM image of: (a) Au NTs, (b) Au NT-AgPt core–shell nanoparticles, (c) Au NT-AgPt core-frame nanoparticles, (d) Au NT-AgPt selective growth nanoparticles, and (e) Au NT-AgPt yolk–shell nanoparticles; the scale bar indicates 50 nm. (f) Corresponding LSPR bands of the particles shown in (a–e).

Optimizing nanostructures that realize both plasmonic and catalytic properties is a hot topic in the field. Several representative structures and their corresponding spectra are summarized and shown in Figure 5. Another interesting observation worth highlighting is the intermediate states of the nanoparticles obtained after 60 min reaction time. The nanostructures obtained after the 60th min had become an Au NT-Ag-Pt core-frame nanoparticle (Figure 5c). The corresponding LSPR band positions were at ∼575 nm, respectively (Figure 5f). The peak was still quite narrow, though, which means that the decay of the surface plasmon resonance (SPR) was less than in the case of the Au NT-Ag-Pt yolk–shell nanoparticles because of the lower Pt content. The damping of plasmon resonance is unfavorable that we like to minimize. First, the dielectric constants of metals are frequency-dependent, wherein the resonance frequency of Ag is higher than that of Au.^47^ The blue-shifting of most particles is caused by the Ag and Pt growth onto the {111} facet of Au NTs and the higher SPR frequency of Ag. In addition, light scattering is an important energy loss mechanism that led to a damping effect because of the strong coupling between electron and electric field at LSPR.^48^ The light scattering is stronger when the size/volume of nanoparticles increases, which is one of the factors that cause the damping effect here. For those core–shell nanostructures, scattering at the metal interface might be another factor contributing to the damping effect. Moreover, the plasmon damping effect of Au–Pt nanoparticles was well revealed in previous studies in both of Au NRs^49,50^ and Au NTs,^34^ which is caused by metal–metal interface damping and charge transfer between Au and Pt.^51^ For the as-synthesized Au-Ag-Pt NPs, compared to Au NTs, the Ag component contributes to the blue shift of LSPR bands and the Pt component leads to the damping effect and results in red-shifting and broadening of LSPR bands. The Au NT-Ag-Pt core-frame structure showed that fewer damping effects might be caused by its frame structure (most of Pt do not directly contact with Au) and lower Pt composition compared to the yolk–shell and core–shell structures. The stronger LSPR indicates a higher potential for being used as a photocatalyst. Lastly, the unique Ag–Pt alloyed nanoframe structures exhibit an extremely high specific surface area, which is advantageous for catalysis.

## Conclusions

In summary, we have demonstrated a diverse strategy to fabricate Au NT-Ag-Pt ternary nanoparticles with various well-designed structures and morphologies, in which AgNO_3_ plays a key role in controlling the morphology of the nanoparticles. The structure of synthesized nanocrystals can be manipulated from yolk–shell to core–shell by varying the concentration of either AgNO_3_ or Au NTs. Moreover, we showed that the Au NT-Ag-Pt yolk–shell structure forms via a growth-Galvanic replacement synergistic mechanism that was revealed by time-resolved UV–vis spectra and STEM images. Notably, Au NT-Ag-Pt core–shell and core-frame structures can be obtained by stopping the growth at a specific reaction time. Compared to the LSPR band of Au NT, the LSPR band of Au NT-Ag-Pt core–shell, yolk–shell, and core-frame nanoparticles showed varying degrees of blue shifts and a decay in intensity, depending on the composition, thickness, and morphology of the Ag-Pt shells. As such, the Au NT-Ag-Pt yolk–shell and core-frame nanostructured particles presented here hold great potential for applications in the oxygen reduction reaction, methanol oxidation reaction in fuel cells, and photocatalytic promotion of the hydrogen evolution reaction.

## Supporting Information Available

More details of experiments, supporting figures, and explanations (PDF)
