# Supplementary material for: Morphology-Controlled Growth of Crystalline Ag–Pt-Alloyed Shells onto Au Nanotriangles and Their Plasmonic Properties
Source: J Phys Chem C Nanomater Interfaces. 2023 Aug 3;127(32):16052–60. doi: 10.1021/acs.jpcc.3c02897 (PMC10441576; doi:10.1021/acs.jpcc.3c02897)
Supplement: Supplementary file 1 — jp3c02897_si_001.pdf [file jp3c02897_si_001.pdf]

Supporting Information

Morphology Controlled Growth of Crystalline Ag-Pt Alloyed Shells onto Au Nanotriangles and their Plasmonic Properties

Xiaobin Xie, [*†‡] *Marijn A. van Huis,* [†] *and Alfons van Blaaderen* [*†‡]

† Soft Condensed Matter, Debye Institute for Nanomaterials Science, Utrecht University, Princetonplein 5, 3584 CC Utrecht, the Netherlands

‡ Present Address: Analytical & Testing Center, Sichuan University, Chengdu 610064, China

* Corresponding Author:

X. Xie E-mail: xb.xie@hotmail.com; A. van Blaaderen E-mail: A.vanBlaaderen@uu.nl.

Contents

1. Determination of Au NTs concentration
2. Reaction conditions of all the synthesis

    2.1 Synthesis of Au NT-AgPt NPs

    2.2 Synthesis of Au NT-AgPt NPs with adding KI

    2.3 Synthesis of Au NT-AuAgPt NPs

    2.4 Synthesis of Au NT-AgPd NPs

3. Key physical parameters of bulk Au, Ag, Pd, and Pt
4. Supporting Figures

1 Determination of Au NTs concentration

Determination of the concentration of Au NTs stock solution. The concentration of Au NTs solution was calculated by measuring the UV-VIS spectra and the equations showed as follow.[S1]

$$\varepsilon = 1.6888 \times 10^{8} e^{5.1742 \times 10^{-3} \times \lambda_{max}} \quad (1)$$

$$C = \frac{A}{\varepsilon l} \quad (2)$$

Where ε is extinction coefficient, λ_{max} is the wavelength of LSPR band of Au NTs, and A is the extinction intensity.

2 Reactions conditions of synthesis

2.1 Synthesis of Au NT-AgPt NPs

Table S1. The reaction conditions used for Au NT-AgPt NPs growth

	**Au NT Solution**	**De-ionized Water**	**0.1 M CTAC**	**10 mM $AgNO_3$**	**10 mM K_2PtCl_4**	**0.10 M AA**	**Figure**
**1**	1.50 mL	7.30 mL	1.00 mL	50 µL	50 µL	100 µL	Fig. 1
**2**	1.50 mL	7.30 mL	1.00 mL	60 µL	50 µL	100 µL	Fig. 2a
**3**	1.50 mL	7.30 mL	1.00 mL	50 µL	50 µL	100 µL	Fig. 2b
**4**	1.50 mL	7.30 mL	1.00 mL	40 µL	50 µL	100 µL	Fig. 2c
**5**	1.50 mL	7.30 mL	1.00 mL	30 µL	50 µL	100 µL	Fig. 2d
**6**	1.50 mL	7.30 mL	1.00 mL	20 µL	50 µL	100 µL	Fig. 2e
**7**	1.50 mL	7.30 mL	1.00 mL	10 µL	50 µL	100 µL	Fig. 2f
**8**	1.00 mL	7.80 mL	1.00 mL	50 µL	50 µL	100 µL	Fig. S3a
**9**	1.50 mL	7.30 mL	1.00 mL	50 µL	50 µL	100 µL	Fig. S3b
**10**	2.00 mL	6.80 mL	1.00 mL	50 µL	50 µL	100 µL	Fig. S3c
**11**	3.00 mL	5.80 mL	1.00 mL	50 µL	50 µL	100 µL	Fig. S3d
**12**	1.50 mL	7.50 mL	1.00 mL	10 µL	10 µL	20 µL	Fig. S8a
**13**	1.50 mL	7.50 mL	1.00 mL	5.0 µL	10 µL	20 µL	Fig. S8b
**14**	1.50 mL	7.50 mL	1.00 mL	2.5 µL	10 µL	20 µL	Fig. S8c
**15**	1.50 mL	7.50 mL	1.00 mL	1.0 µL	10 µL	20 µL	Fig. S8d
**16**	1.50 mL	7.30 mL	1.00 mL	60 µL	/	100 µL	Fig. S4a
**17**	1.50 mL	7.30 mL	1.00 mL	10 µL	50 µL	100 µL	Fig. S4b
**18**	1.50 mL	7.30 mL	1.00 mL	10 µL	20 µL	100 µL	Fig. S4c

**19**	1.50 mL	7.30 mL	1.00 mL	/	50 μL	100 μL	Fig. S4d, Fig. S5
**20**	1.50 mL	7.30 mL	1.00 mL	50 μL	50 μL	100 μL	Fig. S6a
**21**	1.50 mL	7.30 mL	1.00 mL	50 μL	40 μL	100 μL	Fig. S6b
**22**	1.50 mL	7.30 mL	1.00 mL	50 μL	30 μL	100 μL	Fig. S6c
**23**	1.50 mL	7.30 mL	1.00 mL	50 μL	20 μL	100 μL	Fig. S6d
**24**	1.50 mL	7.00 mL	1.00 mL	50 μL	50 μL	500 μL	Fig. S9a
**25**	1.50 mL	7.40 mL	1.00 mL	50 μL	50 μL	100 μL	Fig. S9b
**26**	1.50 mL	7.40 mL	1.00 mL	50 μL	50 μL	40 μL	Fig. S9c
**27**	1.50 mL	7.40 mL	1.00 mL	50 μL	50 μL	20 μL	Fig. S9d

2.2 Synthesis of Au NT-AgPt NPs with adding KI

Table S2. The reaction conditions used for Au NT-AgPt NPs growth.

	**Au NT Solution**	**De-ionized Water**	**0.1 M CTAC**	**10 mM KI**	**10 mM $AgNO_3$**	**10 mM K_2PtCl_4**	**0.10 M AA**	**Figure**
**1**	1.50 mL	7.40 mL	1.00 mL	60 μL	10 μL	20 μL	100 μL	Fig. S10a
**2**	1.50 mL	7.40 mL	1.00 mL	30 μL	10 μL	20 μL	100 μL	Fig. S10b
**3**	1.50 mL	7.40 mL	1.00 mL	15 μL	10 μL	20 μL	100 μL	Fig. S10c
**4**	1.50 mL	7.40 mL	1.00 mL	10 μL	10 μL	20 μL	100 μL	Fig. S10d
**5**	1.50 mL	7.40 mL	1.00 mL	5 μL	10 μL	20 μL	100 μL	Fig. S10e
**6**	1.50 mL	7.40 mL	1.00 mL	/	10 μL	20 μL	100 μL	Fig. S10f

2.3 Synthesis of Au NT-AuAgPt NPs

Table S3. The reaction conditions used for Au NT-AuAgPt NPs growth.

	**Au NT Solution**	**De-ionized Water**	**0.1 M CTAC**	**10 mM $HAuCl_4$**	**10 mM $AgNO_3$**	**10 mM K_2PtCl_4**	**0.10 M AA**	**Figure**
**1**	1.50 mL	7.40 mL	1.00 mL	40 μL	20 μL	30 μL	100 μL	Fig. S14 a, e, m
**2**	1.50 mL	7.40 mL	1.00 mL	30 μL	30 μL	30 μL	100 μL	Fig. S14 b, f, n
**3**	1.50 mL	7.40 mL	1.00 mL	20 μL	40 μL	30 μL	100 μL	Fig. S14 c, g, o
**4**	1.50 mL	7.40 mL	1.00 mL	10 μL	50 μL	30 μL	100 μL	Fig. S14 d, h, p

2.4 Synthesis of Au NT-AgPd NPs

Table S4. The reaction conditions used for Au NT-AgPd NPs growth.

	Au NT Solution	De-ionized Water	10 mM $AgNO_3$	10 mM Na_2PdCl_4	0.10 M AA	Figure
**1**	2.50 mL	7.40 mL	/	50 μL	100 μL	Fig. S15a
**2**	2.50 mL	7.40 mL	10 μL	40 μL	100 μL	Fig. S15b
**3**	2.50 mL	7.40 mL	20 μL	30 μL	100 μL	Fig. S15c
**4**	2.50 mL	7.40 mL	30 μL	20 μL	100 μL	Fig. S15d
**5**	2.50 mL	7.40 mL	40 μL	10 μL	100 μL	Fig. S15e
**6**	2.50 mL	7.40 mL	50 μL	/	100 μL	Fig. S15f

3 Key physical parameters of bulk Au, Ag, Pd, and Pt

A number of physical parameters of Au, Ag, Pd, and Pt are summarized in Table S4, which include their lattice parameters, and the standard reduction potentials.[S2-S5]

Table S5. Key physical parameters of Au, Ag, Pd, and Pt

	Au	Ag	Pd	Pt
**Lattice Parameters**	4.078 Å	4.086 Å	3.891 Å	3.924 Å
**Atomic Radius**	1.442 Å	1.444 Å	1.376 Å	1.387 Å
**Standard Reduction Potentials (V)**	+1.00 ($[AuCl_4]^-$)	+0.80 (Ag^+)	+0.59 ($[PdCl_4]^{2-}$)	+0.76 ($[PtCl_4]^{2-}$)

4 Supporting Figures

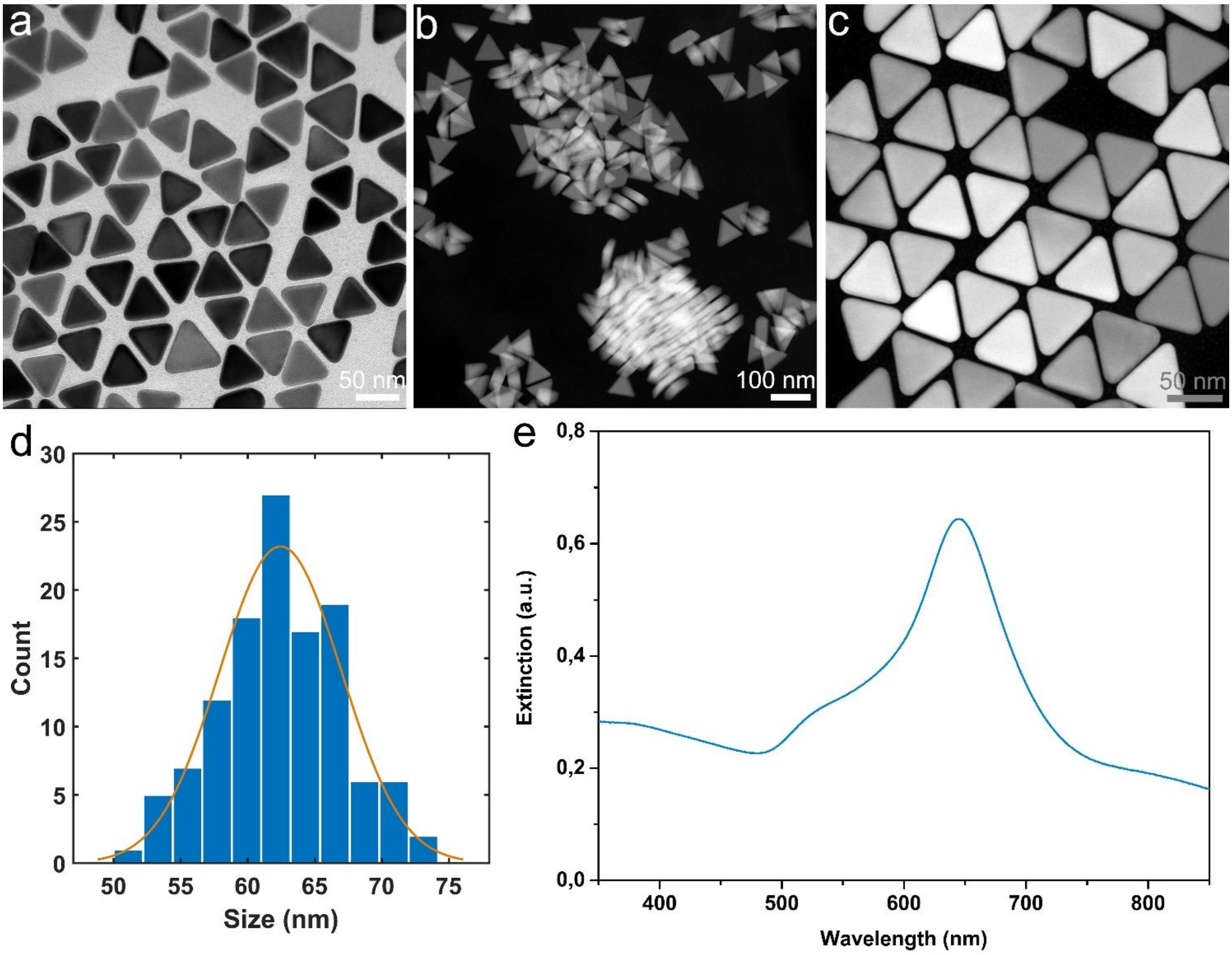

Figure S1. Morphology, size, and main localized surface plasmon resonance (LSPR) band of Au NTs. a) bright-field TEM image of Au NTs; b & c) HAADF-STEM images of Au NTs; d) histogram of edge length of Au NTs; e) UV-VIS spectrum of Au NTs.

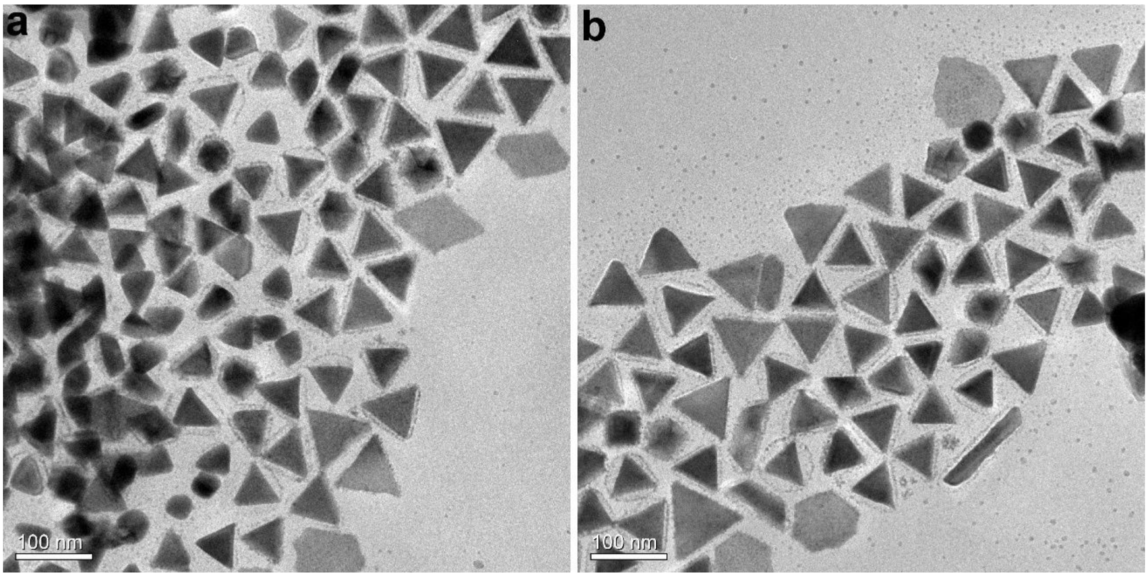

Figure S2. Structural stability of Au NT-AgPt yolk-shell NPs. TEM images of Au NT-AgPt yolk-shell NPs which were placed on a TEM grid for more than six years. The Au NT-AgPt yolk-shell NPs were synthesized and dropped on the TEM grid in September 2016, TEM images showed here were acquired in June 2023. The original morphology of these Au NT-AgPt yolk-shell NPs are showed in Figure 1.

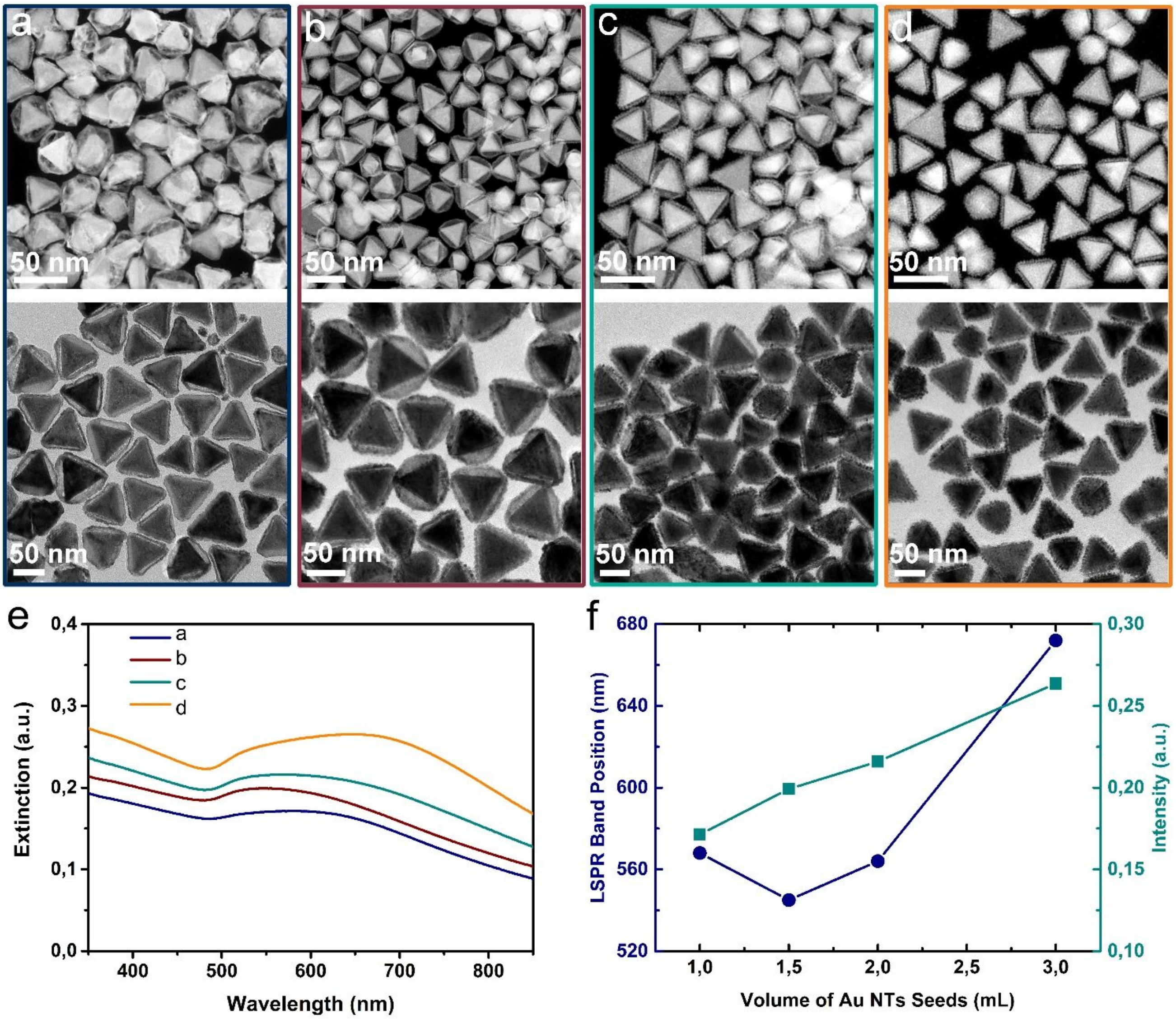

Figure S3. Morphological evolution of Au NT-AgPt NPs synthesized with various concentrations of Au NTs. STEM-HAADF (up) and TEM (down) images indicated Au NT-AgPt NPs acquired as the volume of Au NTs was: a) 1.0 mL, b) 1.5 mL, c) 2.0 mL, d) 3.0 mL. e) UV-Vis spectra and f) LSPR band position of Au NT-AgPt NPs showed in a-d. More details of the synthesis are shown in Table S1.

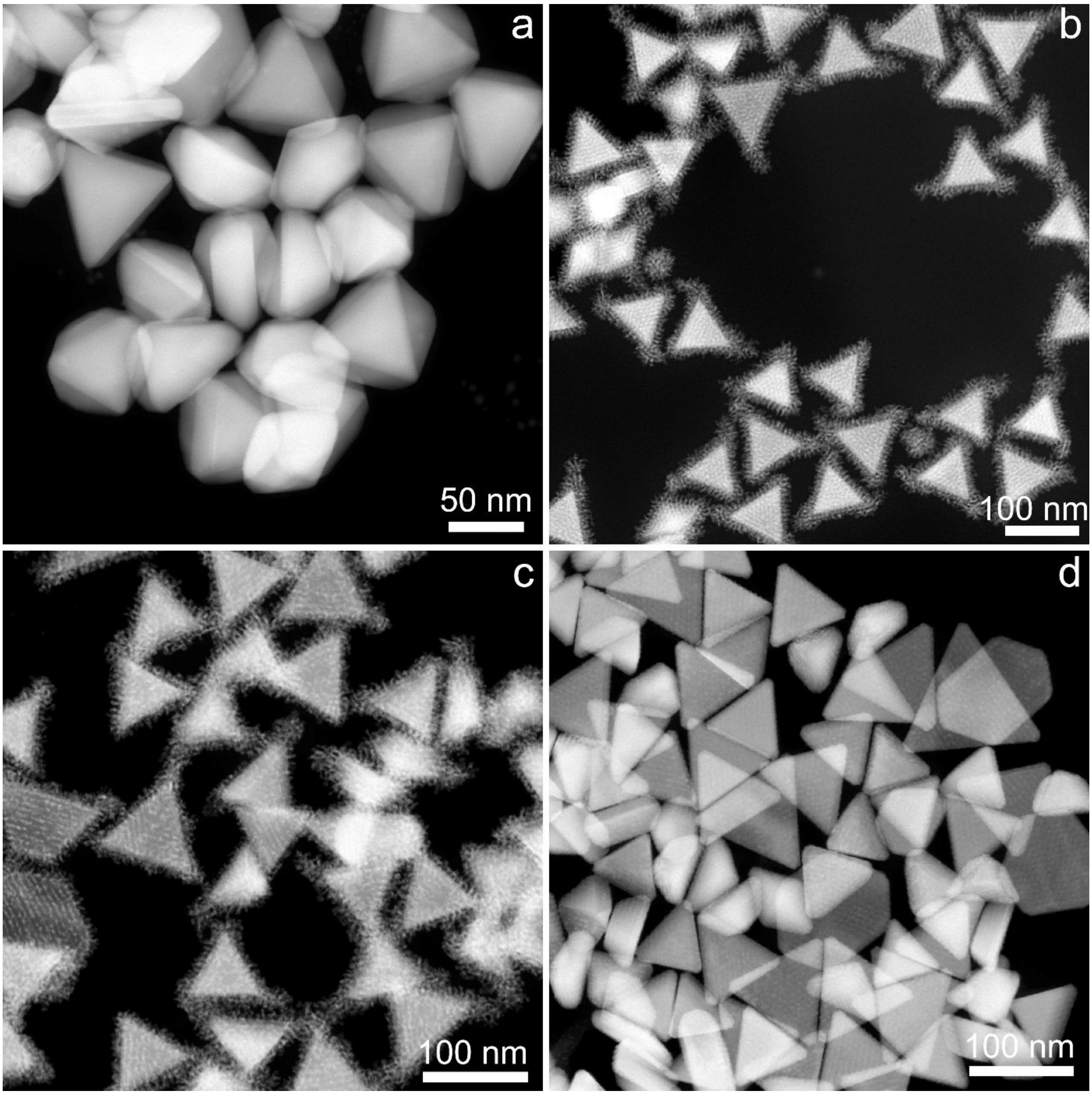

Figure S4. Shape evolution of Au NT-AgPt NPs synthesized by using different $AgNO_3$ and K_2PtCl_4 ratios. STEM-HAADF images indicated NPs acquired as the concentration of $AgNO_3$ and K_2PtCl_4 ratios in the growth solution was: (a) $AgNO_3$ (60 µM)/ K_2PtCl_4 (0 µM), (b) $AgNO_3$ (10 µM)/ K_2PtCl_4 (50 µM), (c) $AgNO_3$ (10 µM)/ K_2PtCl_4 (20 µM), and (d) $AgNO_3$ (0 µM)/ K_2PtCl_4 (50 µM). More details of the synthesis are shown in Table S1.

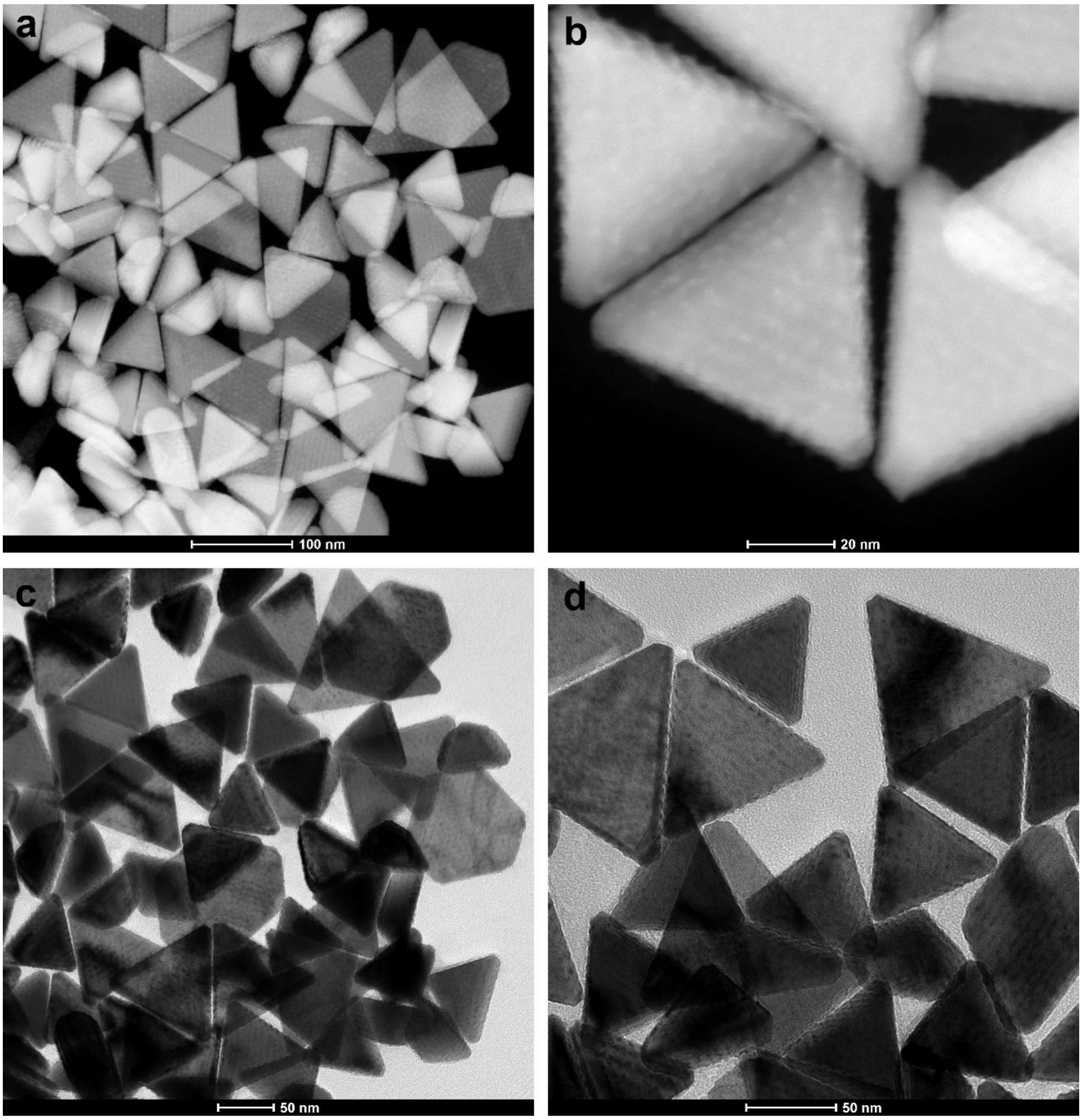

Figure S5. Morphology of Au NT-Pt. (a, b) STEM image of Au NT-Pt NPs, (c, d) TEM image of Au NT-Pt NPs.

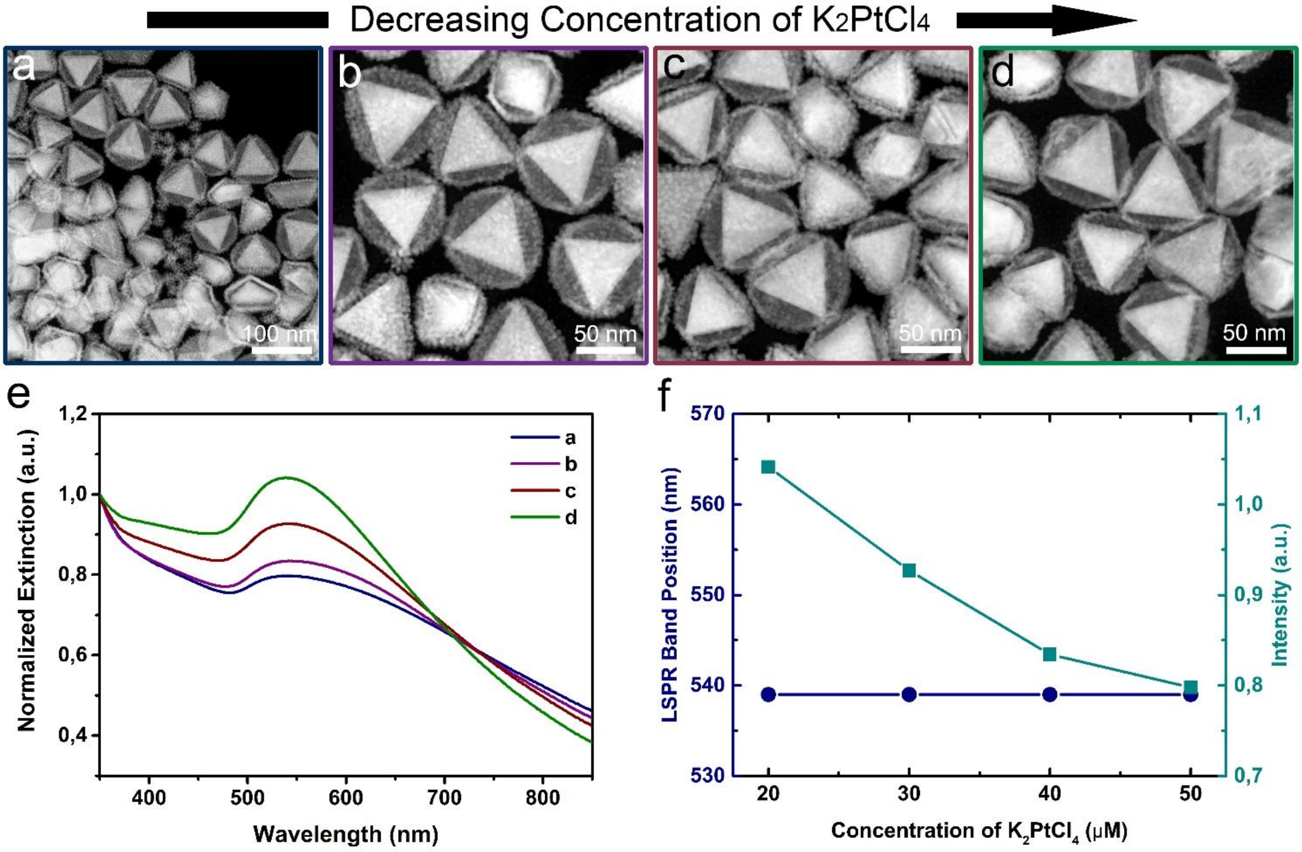

Figure S6. Morphological evolution of Au NT-AgPt NPs synthesized with various concentrations of K_2PtCl_4. STEM-HAADF images indicated Au NTAgPt NPs obtained as the concentration of K_2PtCl_4 was: a) 50 µM, b) 40 µM, c) 30 µM, and d) 20 µM. e) UV-Vis spectra and f) LSPR band position of Au NT-AgPt NPs showed in a-d. More details of the synthesis are shown in Table S1.

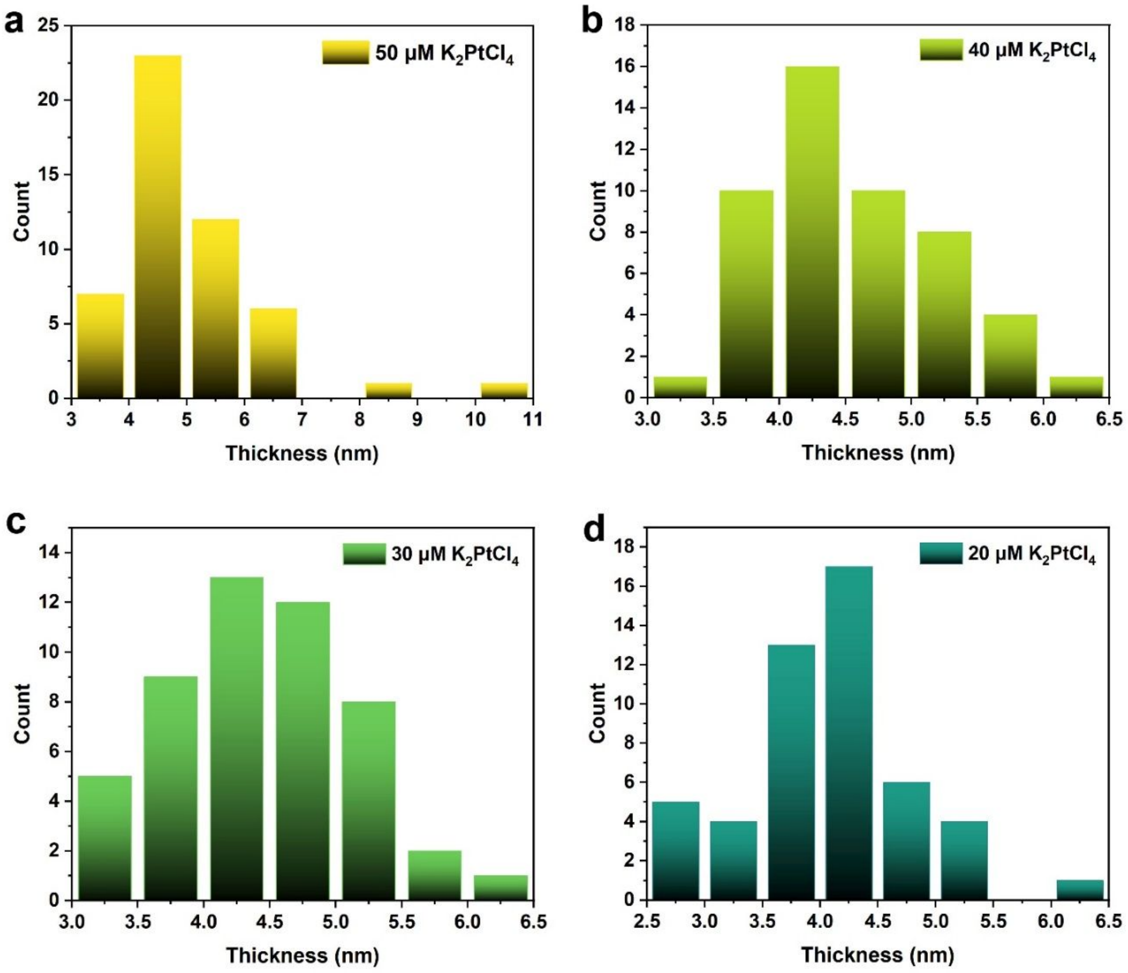

Figure S7. The AgPt shell thickness of Au NT-AgPt NPs synthesized with various concentrations of K_2PtCl_4. Histogram of Ag-Pt shell thickness distributions. The Ag-Pt shell thickness in above Figure was: (a) 5.1±1.2 nm, (b) 4.5±0.7 nm, (c) 4.4±0.7 nm, (d) 4±0.7 nm. Corresponding morphologies are showed in Figure S6.

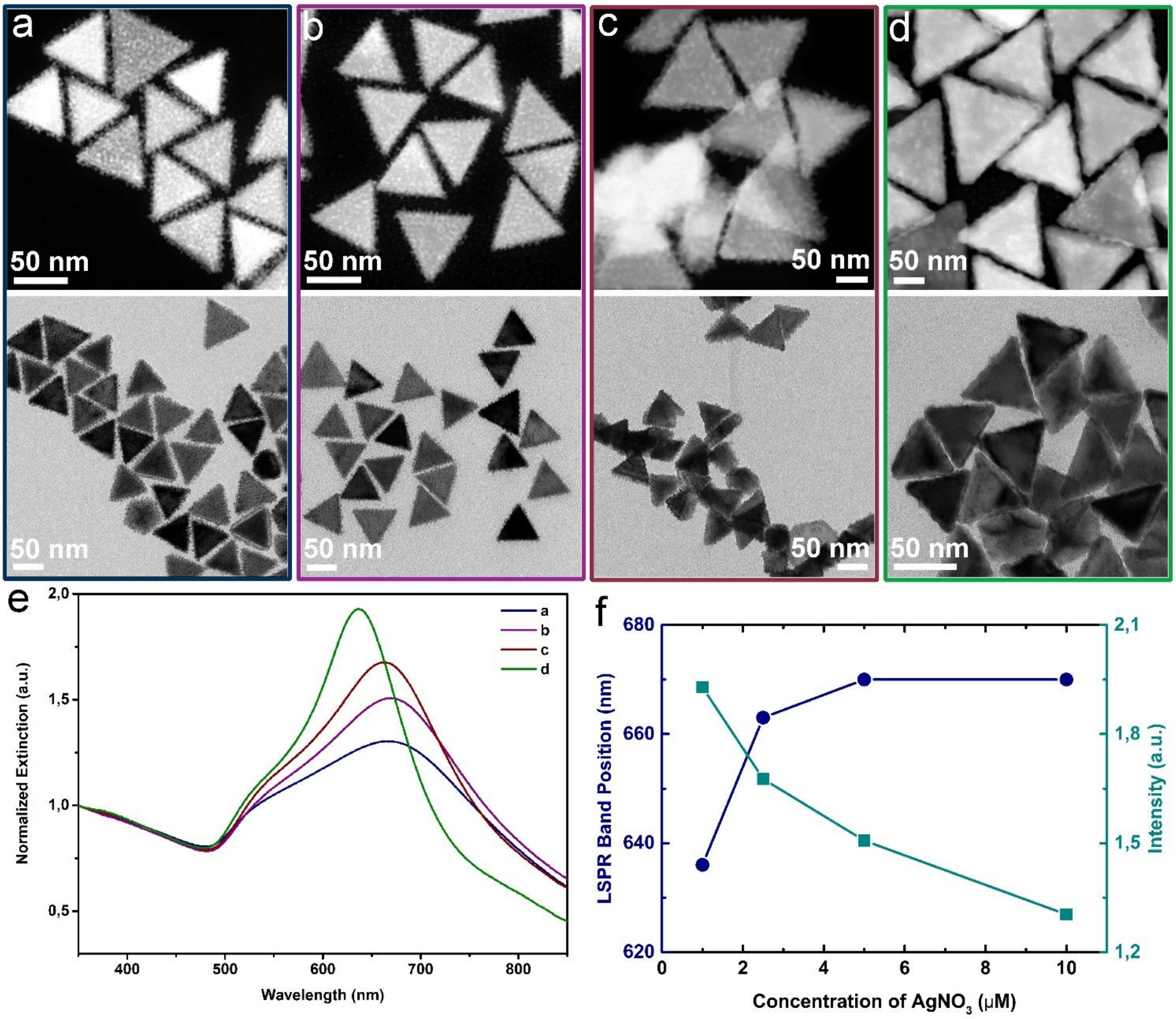

Figure S8. Morphological evolution of Au NT-AgPt NPs for various concentrations of $AgNO_3$. STEM-HAADF (up) and TEM (down) images indicated Au NT-AgPt NPs acquired as the concentration of $AgNO_3$ in the growth solution was: a) 20 μM, b) 15 μM, c) 10 μM, and d) 5 μM. e) UV-Vis spectra and f) LSPR band position of Au NT-AgPt NPs showed in a-d. More details of the synthesis are shown in Table S1.

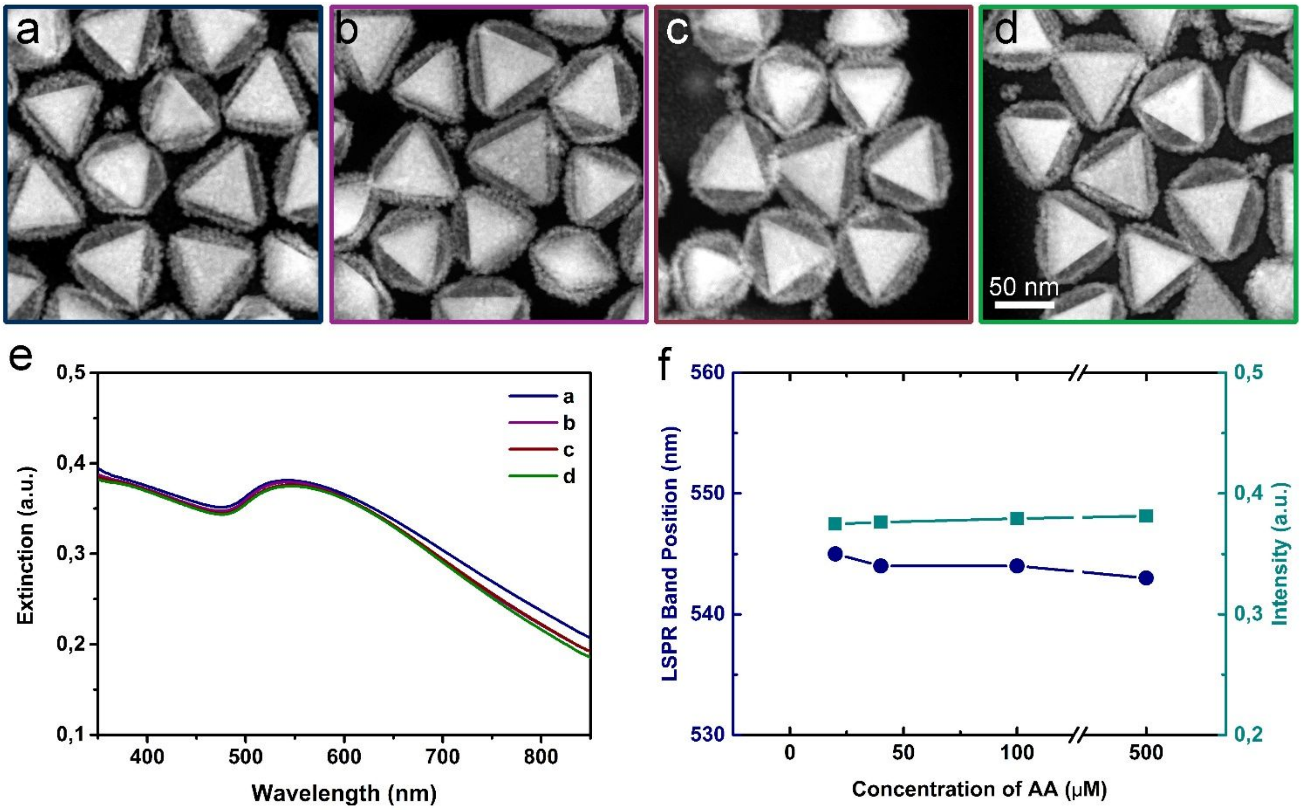

Figure S9. Morphological evolution of Au NT-AgPt NPs synthesized with various concentrations of AA. STEM-HAADF of NPs acquired as the concentration of AA was: (a) 5.00 mM, (b) 1.00 mM, (c) 0.40 mM, and (d) 0.20 mM. (e) UV-Vis spectra and f) LSPR band position of Au NT-AgPt NPs showed in a-d. More details of the synthesis are shown in Table S1.

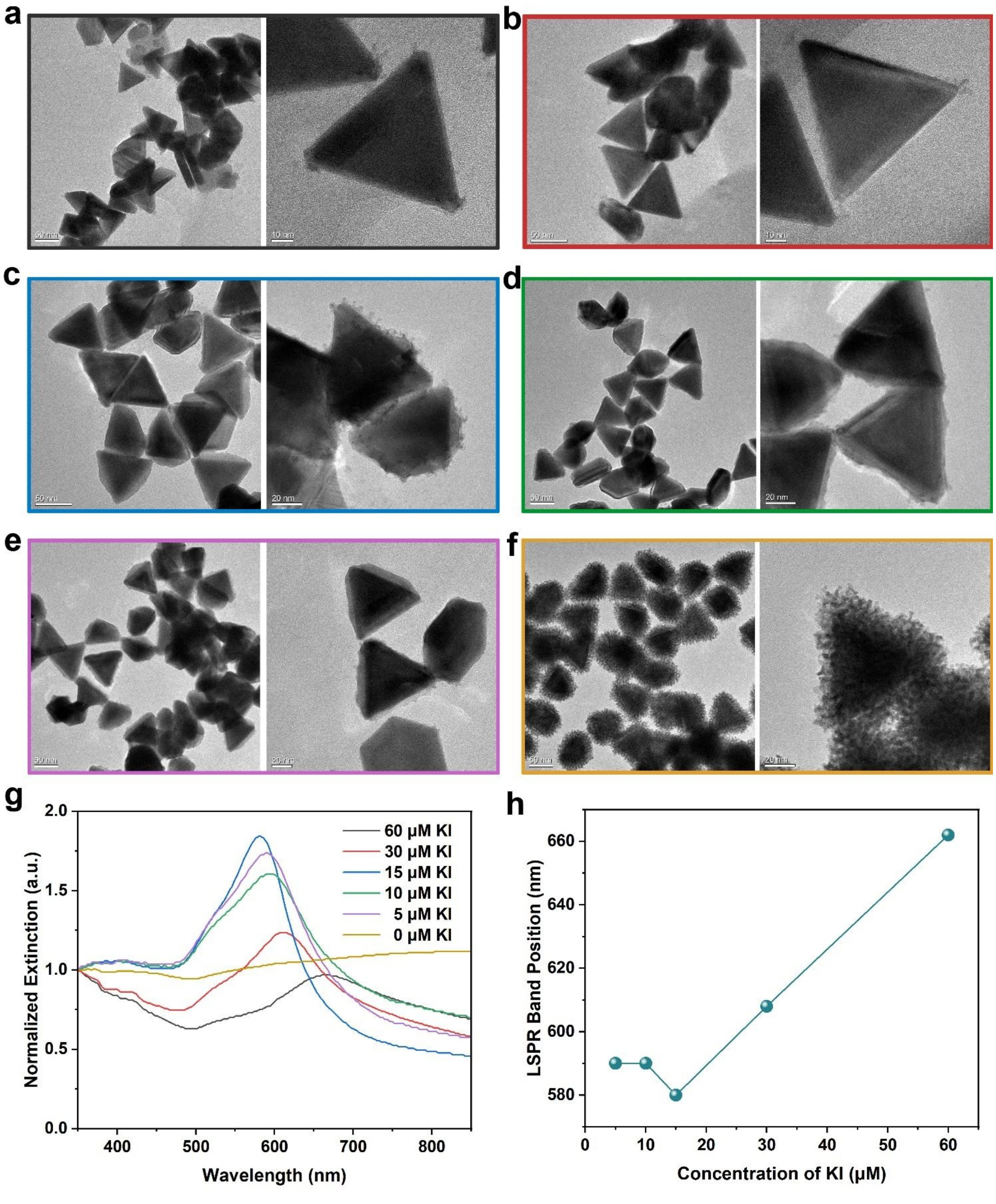

Figure S10. Morphological evolution of Au NT-AgPt NPs synthesized with various concentrations of KI. TEM images of NPs acquired as the concentration of KI was: (a) 60 μM, (b) 30 μM, (c) 15 μM, (d) 10 μM, (e) 5 μM, and (f) 0 μM. (g) UV-Vis spectra and (h) LSPR band position of Au NT-AgPt NPs showed in (a-f). More details of the synthesis are shown in Table S2.

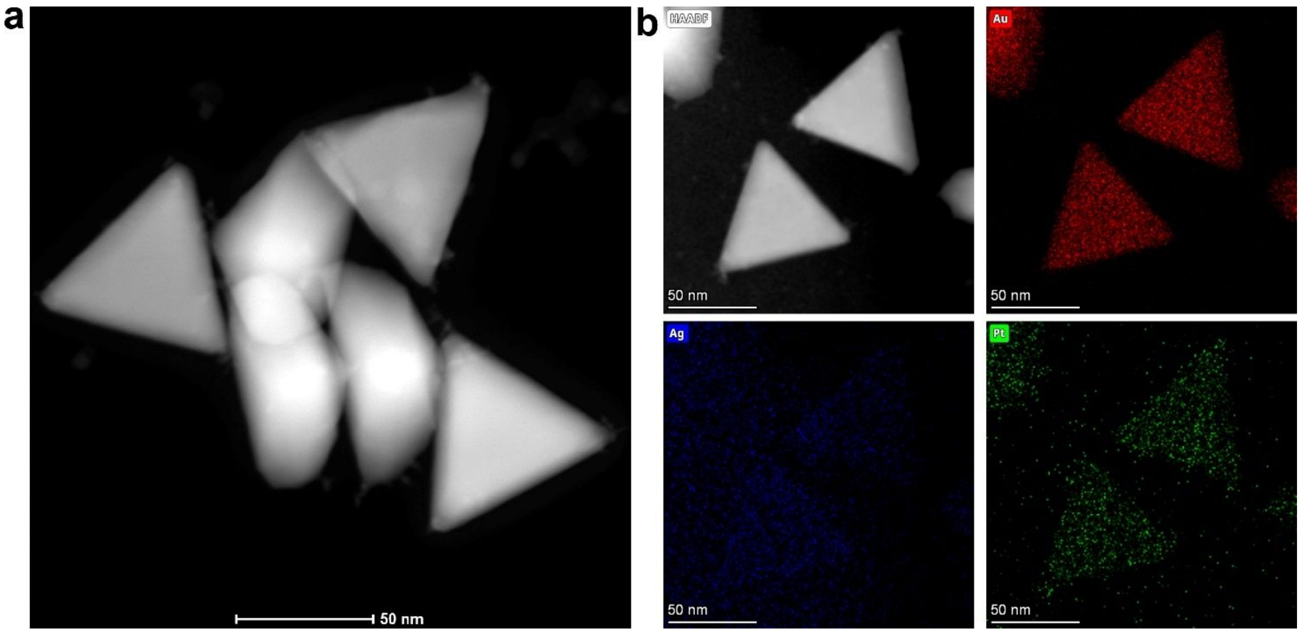

Figure S11. (a) STEM-HAADF image and (b)STEM-EDS elemental maps of Au NT-AgPt site-selective growth NPs which acquired as the concentration of KI was 60 μM.

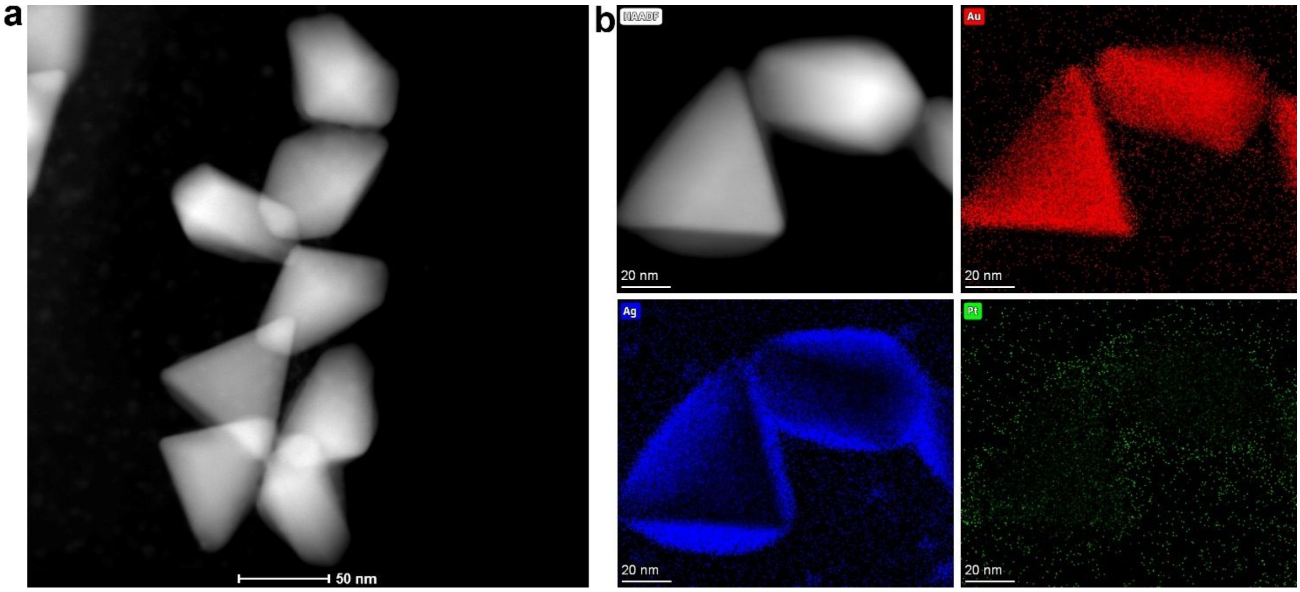

Figure S12. (a) STEM-HAADF image and (b) STEM-EDS elemental maps of Au NT-AgPt NPs which acquired as the concentration of KI was 15 μM.

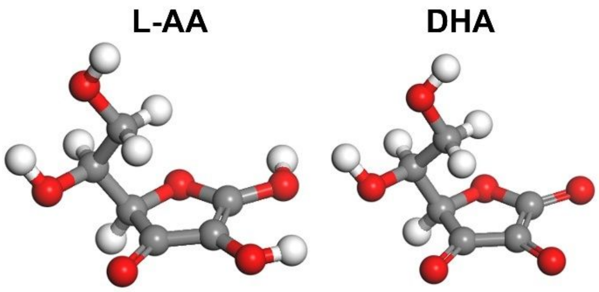

Figure S13. Molecule structure of $C_6H_8O_6$ (L-AA) and $C_6H_6O_6$ (DHA).

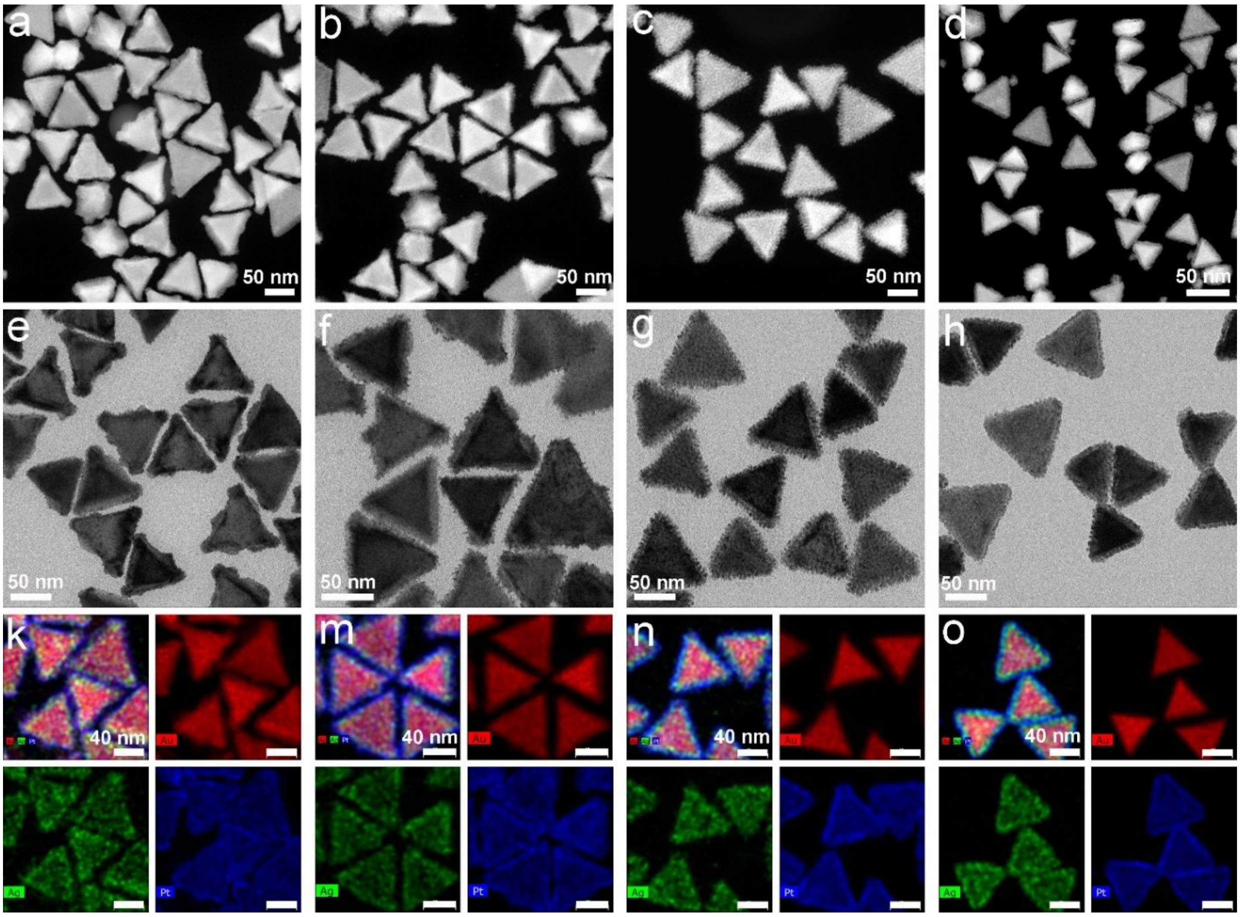

Figure S14. Morphological evolution of Au NT-AuAgPt NPs synthesized with various concentrations of Au and Ag. The concentration of $AgNO_3$ and $HAuCl_4$ ratios in the growth solution was: (a, e, k) $AgNO_3$ (20 μM)/ $HAuCl_4$ (40 μM), (b, f, m) $AgNO_3$ (30 μM)/ $HAuCl_4$ (30 μM), (c, g, n) $AgNO_3$ (40 μM)/ $HAuCl_4$ (20 μM), and (d, h, o) $AgNO_3$ (50 μM)/ $HAuCl_4$ (10 μM).The details of the synthesis are shown in Table S3. The scale bar indicates 50 nm in all STEM images and 40 nm in all STEM-EDS elemental maps.

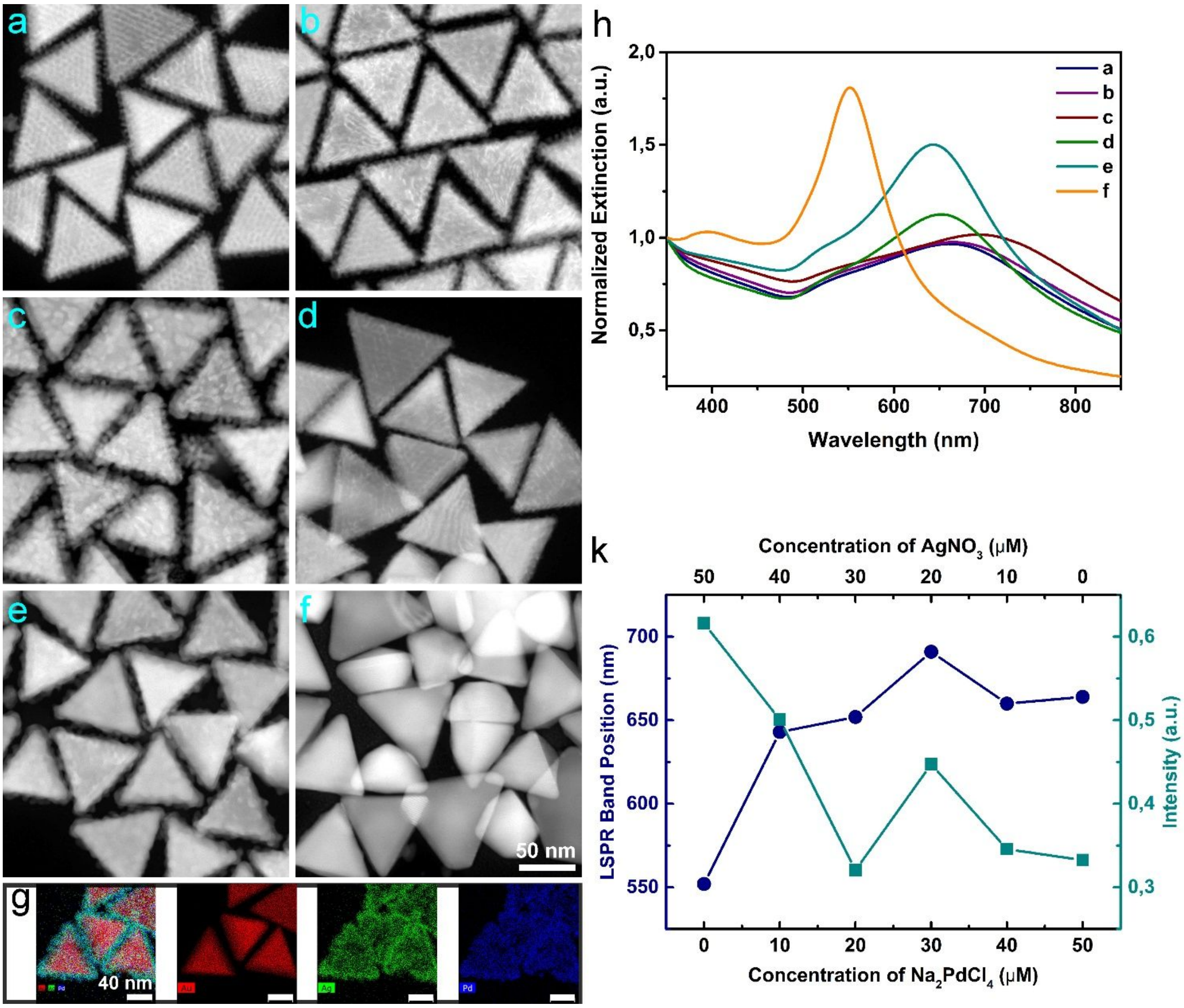

Figure S15. Shape evolution of Au NT-AgPd NPs synthesized while changing the ratio of Pd and Ag precursors. The concentration of $AgNO_3$ and Na_2PdCl_4 ratios in the growth solution was: (a) $AgNO_3$ (0 µM)/ Na_2PdCl_4 (50 µM), (b) $AgNO_3$ (10 µM)/ Na_2PdCl_4 (40 µM), (c, g) $AgNO_3$ (20 µM)/ Na_2PdCl_4 (30 µM), (d) $AgNO_3$ (30 µM)/ Na_2PdCl_4 (20 µM), (e) $AgNO_3$ (40 µM)/ Na_2PdCl_4 (10 µM), and (f) $AgNO_3$ (50 µM)/ Na_2PdCl_4 (0 µM).The details of the synthesis are shown in **Table S4**. The scale bar indicates 50 nm in all STEM images and 40 nm in the STEM-EDS elemental maps.

References:

S1. O'Brien, M. N.; Jones, M. R.; Kohlstedt, K. L.; Schatz, G. C.; Mirkin, C. A., Uniform circular disks with synthetically tailorable diameters: two-dimensional nanoparticles for plasmonics. *Nano Lett.* **2015,** *15* (2), 1012-7.

S2. Johnson, P. B.; Christy, R. W., Optical Constants of the Noble Metals. *Phys. Rev. B* **1972,** *6* (12), 4370-4379.

S3. Fan, F.-R.; Liu, D.-Y.; Wu, Y.-F.; Duan, S.; Xie, Z.-X.; Jiang, Z.-Y.; Tian, Z.-Q., Epitaxial Growth of Heterogeneous Metal Nanocrystals: From Gold Nano-octahedra to Palladium and Silver Nanocubes. *J. Am. Chem. Soc.* **2008,** *130*, 6949–6951.

S4. Bauer, E.; van der Merwe, J. H., Structure and growth of crystalline superlattices: From monolayer to superlattice. *Phys. Rev. B Condens. Matter.* **1986,** *33* (6), 3657-3671.

S5. Xia, Y.; Xia, X.; Peng, H. C., Shape-Controlled Synthesis of Colloidal Metal Nanocrystals: Thermodynamic versus Kinetic Products. *J. Am. Chem. Soc.* **2015,** *137* (25), 7947-66.
